# Plant-Associated *Neoscytalidium dimidiatum*—Taxonomy, Host Range, Epidemiology, Virulence, and Management Strategies: A Comprehensive Review

**DOI:** 10.3390/jof9111048

**Published:** 2023-10-26

**Authors:** Sibel Derviş, Göksel Özer

**Affiliations:** 1 Department of Plant and Animal Production, Vocational School of Kızıltepe, Mardin Artuklu University, Mardin 47000, Turkey; 2 Department of Plant Protection, Faculty of Agriculture, Bolu Abant Izzet Baysal University, Bolu 14030, Turkey

**Keywords:** *Neoscytalidium dimidiatum*, historical overview, intraspecific variation, new synonyms, biology, pathogenicity, control measures, Botryosphaeriaceae

## Abstract

*Neoscytalidium dimidiatum*, a plant- and human-associated fungus, has emerged as a substantial global ecological and agricultural threat aggravated by global warming. It inflicts various diseases, including canker, blight, dieback, leaf spot, root rot, and fruit rot, across a wide spectrum of fruit trees, field crops, shrubs, and arboreal species, with a host range spanning 46 plant families, 84 genera, and 126 species, primarily affecting eudicot angiosperms. Six genera are asymptomatic hosts. *Neoscytalidium dimidiatum* exhibits worldwide distribution, with the highest prevalence observed in Asia and North America, notably in Iran, Turkey, and California. Rising disease prevalence and severity, aggravated by climate change, particularly impact tropical arid places across 37 countries spanning all 7 continents. This comprehensive review encapsulates recent advancements in the understanding of *N*. *dimidiatum*, encompassing alterations in its taxonomic classification, host range, symptoms, geographic distribution, epidemiology, virulence, and strategies for effective management. This study also concentrates on comprehending the taxonomic relationships and intraspecific variations within *N*. *dimidiatum*, with a particular emphasis on *N*. *oculus* and *N*. *hylocereum*, proposing to consider these two species as synonymous with *N*. *dimidiatum*. Furthermore, this review identifies prospective research directions aimed at augmenting our fundamental understanding of host—*N. dimidiatum* interaction.

## 1. Introduction

In the face of escalating environmental changes and global climate fluctuations, the emergence of specific plant pathogens and the diseases they induce have become a pressing concern. Within this context, the monospecific plant pathogenic genus *Neoscytalidium*, represented by *N*. *dimidiatum*, has garnered substantial attention due to its remarkable adaptability and aggressive nature [1,2]. This pathogen poses a formidable threat as it gives rise to epidemics affecting a diverse array of plant species, manifesting as canker and dieback diseases that impact economically, industrially, forestally, and ornamentally important trees and shrubs. This adaptability, coupled with its aggressive tendencies, renders it a significant menace to the global agriculture and horticulture sectors. While *Neoscytalidium* is predominantly recognized as a phytopathogen, it also engenders a range of clinical conditions in humans. It affects individuals with underlying predispositions as well as those seemingly devoid of health concerns [3,4].

The pathogen enters host plant tissues through pre-existing wounds, giving rise to a spectrum of symptoms. Notably, host plants undergoing abiotic stress tend to exhibit intensified disease manifestations post-infection. In the realm of perennial plants like dragon fruits [5,6,7,8,9,10,11,12,13,14,15,16,17,18], citruses [19,20], grapevines [21,22,23,24,25,26], pines [27,28], stone fruits [29,30,31,32,33], *Ficus* spp. [34,35,36,37,38,39,40,41], pistachios [42], and willows [43,44], the symptomatic expression is particularly conspicuous, leading to yield reduction and shortened lifespans. In the context of dragon fruit canker, a devastating affliction affecting dragon fruit plants worldwide, the initiation of fungal infection ensues with the development of appressoria on the surface, followed by direct penetration into epidermal cells [45]. The transmission mechanisms of *N*. *dimidiatum* encompass seeds, propagation materials, soil, and airborne dispersal, with a marked tendency to persist within soil alongside infected debris [39,45,46,47,48,49].

Characterized by *Scytalidium*-like arthric chains of dark conidia and *Fusicoccum*-like conidia in conidiomata, *N*. *dimidiatum* is distinct [2,50,51,52,53]. Since the presence of ascospore-producing ascoma has not been documented, both coelomycetous and hyphomycetous morph conidia from diseased plant parts may serve as the primary source of *Neoscytalidium*-caused diseases [39,45,49]. However, the epidemiology, including the seasonal dynamics of various spore types, remains insufficiently elucidated. The taxonomic journey of *N*. *dimidiatum* has been marked by significant discoveries and revisions. Initially recognized as distinct species, such as *N*. *novaehollandiae* and *N*. *orchidacearum*, recent research endeavors have led to their consolidation under the species of *N*. *dimidiatum* [2]. However, the taxonomic landscape continues to evolve, with the emergence of novel fungal species such as *N*. *oculus* [54] and *N*. *hylocereum* [55], underscoring the intricate genetic diversity within this fungal group.

The adaptability of thermotolerant *Neoscytalidium* to diverse environmental conditions, including elevated temperatures and drought periods, likely contributes to its heightened virulence and extensive global distribution. To provide a comprehensive understanding of *N*. *dimidiatum*, this review amalgamates existing research while delving into novel insights across its attributes, epidemiology, virulence, and prospective management strategies. By enriching our comprehension of *N*. *dimidiatum*’s significance within the Botryosphaeriales order, this review augments our knowledge of this pathogen and its multifaceted impacts.

## 2. Taxonomy and Classification of *Neoscytalidium dimidiatum*

### 2.1. Historical Changes in Neoscytalidium dimidiatum Taxonomy

The taxonomic classification of *N*. *dimidiatum* has a turbulent history due to the fact that it generates two distinct asexual states known as synanamorphs, which have been described by multiple authors. The coelomycetous morph produces pycnidia with conidia that resemble *Fusicoccum*-like conidia produced in pycnidia, while the hyphomycetous morph produces arthric chains of conidia in the aerial mycelium, giving it a powdery appearance that resembles *Scytalidium*-like conidia [56,57,58]. Derived from the arthric synanamorph, the initial description of *N*. *dimidiatum* occurred when Penzig named it *Torula dimidiata* Penz. in 1882 [59]. Subsequently, another synanamorph of this species, categorized as a coelomycete, was identified by Nattrass [56] in 1933 and designated as *Hendersonula toruloidea*. This recognition came approximately fifty years after the initial classification by Penzig and was observed in pome and stone fruit trees. Wilson [60] proposed the name *Exosporina fawcetti* as the causal agent of a sudden wilt affecting Persian walnut trees (*Juglans regia*) in California. This assignment was grounded in the resemblance to a comparable incident reported by Fawcett [RAM., xv, p. 574] in 1923, wherein the incident took place within the same county and involved grapefruit and orange trees displaying similar symptomatic manifestations. Campbell and Mulder [61] introduced the new species *Scytalidium hyalinum* as the causal agent for the same clinical lesions previously associated with *H*. *toruloidea*. In the research conducted by Sutton and Dyko [57], a taxonomic reorganization was undertaken. They reclassified *Dothiorella mangiferae*, *Fusicoccum eucalypti*, *Hendersonula agathidis*, *Hendersonula cypria*, and *Hendersonula toruloidea* into *Nattrassia mangiferae*. Additionally, they introduced the name ‘*Scytalidium dimidiatum*’ to designate the corresponding mycelial synanamorph, drawing from the nomenclature of *Torula dimidiata*. This study also encompassed *Torula dimidiata*, *Exosporina fawcettii*, and *Scytalidium lignicola*, which were incorporated into the synonymy of *Scytalidium dimidiatum* based on shared mycelial synanamorphic characteristics. Subsequently, Farr et al. [58] conducted phylogenetic analysis using internal transcribed spacer region (ITS) and beta-tubulin gene (*tub2*) sequences, which led to the determination that *Nattrassia mangiferae* and *Scytalidium dimidiatum* should be reclassified under *Fusicoccum*, resulting in the name *Fusicoccum dimidiatum*. Additionally, they assigned the specific isolates of *N*. *mangiferae* responsible for inducing cankers on Pacific madrone trees as *Fusicoccum arbuti*. Through DNA sequencing, a definitive differentiation has been established between genuine *N*. *mangiferae* isolates and *F*. *arbuti*. However, Slippers et al. [62] conducted a study on isolates identified as *Dothiorella mangiferae* (*Nattrassia mangiferae*) obtained from mango trees in Australia and determined that they actually belonged to *Fusicoccum*. Consequently, they introduced the name *Fusicoccum mangiferae* (now classified as *Neofusicoccum mangiferae*) for this group of isolates. They also did not observe the *Scytalidium*-like synanamorph, which is consistent with the observations made by Sutton and Dyko [57] and Sydow et al. [63]. Therefore, the synonymy of *H*. *toruloidea* (which possesses a *Scytalidium*-like synanamorph) with *F*. *mangiferae* (which does not exhibit a *Scytalidium*-like synanamorph) was rejected. In a taxonomic revision of the Botryosphaeriaceae, Crous et al. [50] concluded that *Scytalidium* is polyphyletic and proposed the genus *Neoscytalidium*, accommodating *S*. *dimidiatum* as *N*. *dimidiatum*. Their study additionally unveiled the distinct classification of *S*. *dimidiatum* and *Scytalidium*’s type species, *Scytalidium lignicola*, as they belong to separate classes. Pavlic et al. [51] identified a fungal species as *N*. *novaehollandiae*, which was collected in Western Australia in July 2006, particularly from *Crotalaria medicaginea*. There have been suggestions that *S*. *dimidiatum* and S. *hyalinum* may be conspecific, proposing the name *N*. *dimidiatum* var. *hyalinum* [64]. In their study, Phillips et al. [52] classified the species as *N*. *hyalinum* and employed a multi-locus tree encompassing ITS, translation elongation factor 1-alpha gene (*tef1*), *tub2*, large subunit (of ribosomal RNA) gene (LSU), and small subunit (of ribosomal RNA) gene (SSU) sequences for its analysis within the Botryosphaeriaceae family. Huang et al. [53] identified a coelomycete in Thailand resembling asexual morphs within Botryosphaeriaceae. Through morphological and phylogenetic analysis, the strain was revealed as a new species, *Neoscytalidium orchidacearum*. Considering the phylogenetic similarity between *S*. *hyalinum* and *N*. *dimidiatum* and the fact that *S*. *hyalinum* is the older name, they proposed transferring *S*. *hyalinum* to the new genus *Neoscytalidium* and synonymizing *N*. *dimidiatum* [53]. Within the *Neoscytalidium* genus, Zhang et al. [2] conducted a comprehensive analysis by sequencing ITS, *tef1*, and *tub2* genes across various *Neoscytalidium* spp. As a result, all examined species were determined to belong to *N*. *dimidiatum* (Penz.) Crous & Slippers. Additionally, *N*. *novaehollandiae* and *N*. *orchidacearum* were found to be synonymous with *N*. *dimidiatum*. To support this taxonomic assignment, the nucleotide similarities between the ex-type culture of *N*. *dimidiatum* and the sequences derived from the ex-type cultures of *N*. *novaehollandiae* and *N*. *orchidacearum* were compared. Specifically, the ITS gene showed 489 out of 492 matching positions (99.39%) with *N*. *novaehollandiae* and 486 out of 492 matching positions (98.78%) with *N*. *orchidacearum*. Regarding the *tef1* gene, a similarity of 185 out of 187 positions (98.93%) was observed with *N*. *novaehollandiae*, while no *tef1* sequence was available for *N*. *orchidacearum*. Moreover, the *tub2* gene exhibited complete identity, with 100% sequence similarity at all 358 positions between *N*. *dimidiatum* and *N*. *novaehollandiae*, while no *tub2* sequence was available for *N*. *orchidacearum*. Zhang et al. [2] also highlight the significant utility of the *tef1* gene, which encodes translation elongation factor 1-alpha, in effectively distinguishing between various *Neoscytalidium* species.

### 2.2. Significance of Resolving Neoscytalidium Species Concepts

Resolving *Neoscytalidium* species concepts is crucial for addressing essential concerns related to polyphagous pathogen identification and control. It facilitates the implementation of control measures like crop rotation and mixed plantations; enhances our understanding of disease dynamics, allows for resistance monitoring; and supports epidemiological studies and research in fungal biology, ecology, and genetics. Ultimately, taxonomic clarity empowers comprehensive disease management and deepens our insights into disease dynamics.

The designation of a specific population or taxonomic group as a new fungal species is a central topic in taxonomic discussions [65]. Currently, the scientific community recognizes more than 30 distinct species concepts, although only a subset of these concepts is widely used in fungal taxonomy [66]. Each concept has its own advantages and disadvantages, and there is no universally agreed-upon standard. The process of taxonomic consolidation observed here mirrors the intricate historical complexities surrounding the classification of *Neoscytalidium*. This history has been marked by taxonomic disagreements and shifts in species boundaries, leading to challenges in understanding the species’ unique biology.

The use of both morphological and DNA-based methodologies has sometimes resulted in the excessive subdivision of *N*. *dimidiatum* into multiple taxa [50,51,52,53,56,57,58,59,60,61,64]. Conversely, other methodologies have grouped various taxa into a single species category [2,50,57,58,62]. Addressing these challenges requires the application of multiple genetic markers and coalescence-based methodologies, offering the potential for more precise and reliable insights into the complex taxonomic relationships within the *Neoscytalidium* genus.

In conclusion, the genetic investigation conducted by Zhang et al. [2] holds significant relevance in elucidating the taxonomic intricacies within the *Neoscytalidium* genus. Their integration of multiple gene loci brings clarity to the consolidation of species, with an emphasis on the role of *tef1* in distinguishing *Neoscytalidium* species. Collectively, these studies provide invaluable insights into the taxonomy and delineation of species within *Neoscytalidium*. The taxonomic classification within the *Neoscytalidium* genus remains an ongoing area of investigation, and a more comprehensive understanding necessitates further sequencing of additional genetic markers for representative strains of different species. Achieving a conclusive taxonomic resolution for this genus requires continued research efforts.

### 2.3. Molecular Evidence for Synonymy and Intraspecific Variation in Neoscytalidium dimidiatum

Precise taxonomic classification hinges upon a thorough comprehension of the genetic inter-relationships within closely related fungal species. Evidently, previously acknowledged taxonomic entities, such as *N. novaehollandiae* and *N. orchidacearum*, have undergone consolidation into *N. dimidiatum*, as elucidated by Zhang et al. [2]. Nevertheless, the taxonomic reassignment of *N. oculus* and *N. hylocereum* to *N. dimidiatum* remains pending and necessitates an in-depth inquiry for resolution.

In this context, it is pertinent to mention the work of Calvillo-Medina et al. [54], who introduced *N*. *oculus*, a novel fungal species linked to human keratitis in Latin America. Their taxonomic assignment, however, did not incorporate the *tef1* and *tub2* genes. Instead, they relied on a phylogenetic analysis of concatenated ITS and LSU ribosomal DNA sequences, along with morphological observations, to establish this new species. Another notable instance is the work of Wonglom et al. [55], where a distinct fungal pathogen, *N*. *hylocereum*, was identified. This discovery was achieved through a combined approach involving morphological assessments and molecular analyses. The DNA sequences derived from the ITS, *tef1*, and *tub2* loci distinctly positioned these newly discovered isolates in a separate clade.

This part of the review aims to elucidate the taxonomic relationships and intraspecific variations within *Neoscytalidium* species, with a particular focus on *N*. *oculus* and *N*. *hylocereum*. These two species exhibit morphological similarities while posing challenges for precise species differentiation.

**Figure 1:**
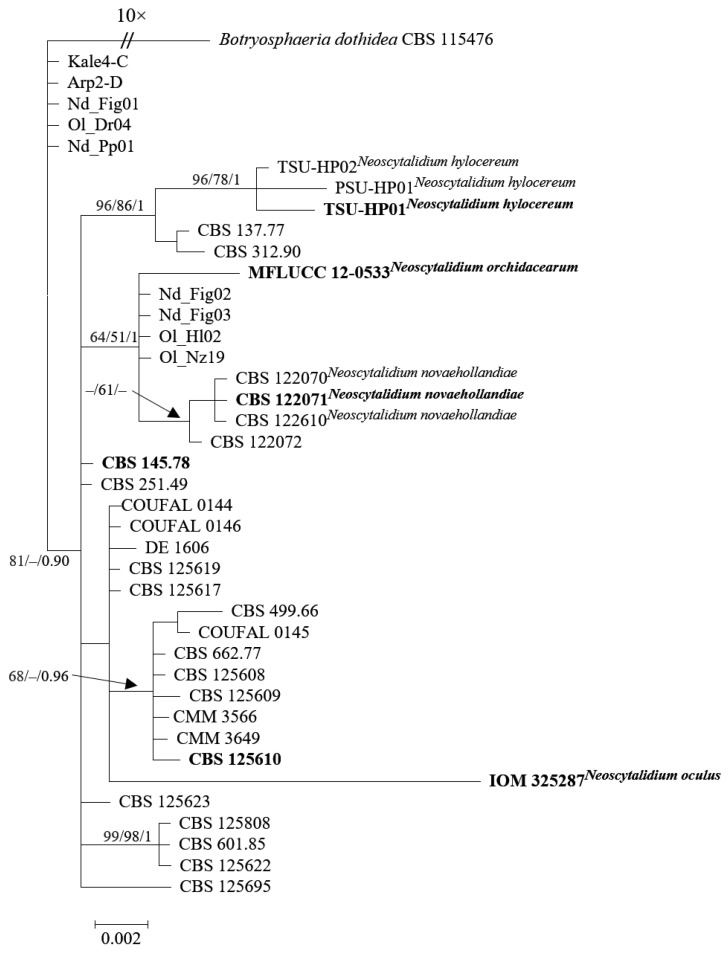
Phylogenetic tree of *Neoscytalidium* resulting from a Bayesian analysis of the combined ITS, *tef1*, *tub2* sequence alignment. Maximum Likelihood and Maximum Parsimony bootstrap support values (ML/MP-BS > 50%) and Bayesian posterior probabilities (PP > 0.90) are shown at the nodes. Ex-type strains and taxonomic novelties are indicated in bold font. The last accepted species names are shown in superscript where species were synonymized in this study; species names on which the name of the clade is based are in bold superscript. The tree was rooted to *Botryosphaeria dothidea* (CBS 115476).

To address this challenge, we employed molecular analyses to clarify their taxonomic designations (Figure 1). The analysis of ITS, *tef1*, and *tub2* loci (Table S1) was conducted using sequences obtained from the GenBank nucleotide database. Multiple sequence alignments were performed using the MAFFT v.7 online interface [67] and subsequently manually edited in MEGA X [68]. Phylogenetic trees were constructed using Maximum Likelihood (ML), Maximum Parsimony (MP), and Bayesian analysis (BA). ML analysis utilized RAxML-HPC BlackBox v. 8.2.10 [69], MP analysis was conducted with PAUP v. 4.0b10 [70], and Bayesian analysis employed MrBayes 3.2.7 [71]. The resulting trees were visualized using FigTree v. 1.4.2 (http://tree.bio.ed.ac.uk/software/figtree, accessed on 22 August 2023). *Botryosphaeria dothidea* (CBS 115476) served as an outgroup to root the tree.

The exploration of *N*. *oculus* and *N*. *dimidiatum* isolates began with a detailed investigation of their genetic makeup through LSU and ITS sequencing. Surprisingly, the LSU sequence demonstrated a remarkable genetic similarity of up to 100%, suggesting an intimate genetic connection. This finding, however, is juxtaposed by discernible variations in the ITS sequence. Particularly, disparities emerged within specific regions: the terminal 11 bases of the SSU, the first 24 bases of ITS1, and the terminal 43 bases of the ITS2 region. The discrepancies, encompassing 65 differing bases, also include the critical 22 bases crucial for binding to LSU rRNA. It is noteworthy that BLAST analysis identified self-overlapping occurrences exclusively within the GenBank database for these specific regions, implying potential inaccuracies in the sequence data input.

Further exploration focused on the *tef1* sequences of *N*. *hylocereum* isolates, revealing a substantial similarity ranging from 99.61% to 100% when compared to *tef1* sequences of *N*. *dimidiatum*. This genetic resemblance establishes a foundational criterion for their taxonomic classification. However, the diversity observed in ITS sequences, specifically within the terminal 13 bases of the SSU and the initial 24 bases of ITS1, suggests that relying solely on a single gene sequence might be insufficient for precise classification.

Delving deeper into the genetic makeup, analysis of the *tub2* sequences revealed genetic disparities among the isolates, primarily rooted in variances within the initial 16 nucleotides of these sequences. The outcomes of BLAST analysis conducted on these sequences brought forth instances of self-matching solely within the NCBI GenBank nucleotide database, further supporting the notion of potential errors in sequence inputs.

In a collective summary, the comprehensive molecular analyses presented in this study provide strong evidence to support the proposition of synonymy between *N*. *oculus* and *N*. *hylocereum* with *N*. *dimidiatum*. While genetic similarities in specific regions are apparent, the highlighted differences within key gene regions underscore the intricate nature of their genetic relationships. The revelation of a unique ITS clade within the Turkish isolates, notably distant from reference isolates, highlights the critical need for precise taxonomic classification, especially in light of differences across multiple gene regions.

In conclusion, the comprehensive assessments of LSU, ITS, *tef1*, and *tub2* sequences offer strong evidence supporting the classification of *N*. *oculus* and *N*. *hylocereum* as synonyms of *N*. *dimidiatum*. The observed genetic resemblances are accompanied by significant differences within crucial gene regions. The case of *N*. *oculus* further emphasizes the necessity of employing diverse gene regions for accurate species identification, shedding light on the intricate taxonomic complexity in the fungal realm.

Moreover, the inclusion of previously documented Turkish isolates from diverse hosts enhances the depth of our phylogenetic exploration concerning *N*. *dimidiatum* isolates, revealing a distinctive ITS clade embedded within the species. Through their application of molecular phylogenetic methodologies involving ITS, *tef1*, and *tub2* sequence alignments, Zhang et al. [2] marked off the examined Turkish isolates, specifically Kale4-C and Arp2-D, as a distinct and recognizable group separate from the broader population of *Neoscytalidium* isolates, designating this particular cluster as *Neoscytalidium* sp.1. Even though Güney et al. [49]’s results agreed with the observation of this phylogenetic divergence among isolates from different chickpea cultivation sites, their subsequent statistical analysis did not show any significant differences in the size of spores produced both as arthroconidia and within conidiomata among these isolates that were in a separate clade. The clear separation of specific Turkish isolates into a separate cluster, evident when comparing them to the larger collection of recognized *Neoscytalidium* isolates in phylogenetic analyses relying on multi-locus sequences, strengthens the understanding that this divergence is best explained as a characteristic of intraspecies polymorphism. This interpretation holds more weight than considering it as sufficient evidence to classify these isolates as distinct taxonomic entities.

### 2.4. Current Species Name

*Neoscytalidium dimidiatum* (Penz.) Crous & Slippers [2,50].

### 2.5. Classification

Kingdom Fungi; Phylum Ascomycota; Subphylum Pezizomycotina; Class Dothideomycetes; Subclass Incertae sedis; Order Botryosphaeriales; Family Botryosphaeriaceae; Genus *Neoscytalidium*.

### 2.6. Synonyms and Basionyms

1. *Torula dimidiata* Penz. [59].
2. *Hendersonula toruloidea* Nattrass [56].
3. *Exosporina fawcettii* E.E. Wilson [60].
4. *Scytalidium hyalinum* C.K. Campb. & J.L. Mulder [61].
5. *Scytalidium dimidiatum* (Penz.) B. Sutton & Dyko [57].
6. *Fusicoccum dimidiatum* (Penz.) D.F. Farr [58].
7. *Neoscytalidium dimidiatum* (Penz.) Crous & Slippers [50].
8. *Neoscytalidium novaehollandiae* Pavlic, T.I. Burgess & M.J. Wingf. [51].
9. *Neoscytalidium dimidiatum* var. *hyalinum* (C.K. Camp. & J.L. Mulder) Madrid, Cano, Stchigel & Guarro [64].
10. *Neoscytalidium hyalinum* (C.K. Campb. & J.L. Mulder) A.J.L. Phillips, Groenewald & Crous [52].
11. *Neoscytalidium orchidacearum* S. K. Huang, N. Tangthirasunun, J. C. Kang & K. D. Hyde [53].
12. *Neoscytalidium oculus* (*Neoscytalidium oculi* in MycoBank) J. Mena, Raymundo & Bautista-de-Lucio (the taxonomic status of this species was consolidated as a synonym in the present investigation).
13. *Neoscytalidium hylocereum* Kheawleng, Intaraa-nun & Rodkaew (the taxonomic status of this species was consolidated as a synonym in the present investigation).

### 2.7. Rejected Synonyms

1. *Dothiorella mangiferae* Syd. & P. Syd. (1916) [57].
2. *Fusicoccum eucalypti* Sousa da Câmara (1929) [57].
3. *Hendersonula cypria* Nattrass (1937) [57].
4. *Hendersonula agathidis* H.E. Young (1948) [57].
5. *Nattrassia mangiferae* (Syd. & P. Syd.) B. Sutton & Dyko [57].
6. *Fusicoccum mangiferae* (Syd. & P. Syd.) Johnson, Slippers & M.J. Wingf. [62].
7. *Scytalidium lignicola* Pesante [50].

Note: The first six species listed above are currently classified as *Neofusicoccum mangiferae* (Syd. & P. Syd.) Crous, Slippers & A.J.L. Phillips [50].

## 3. Identification

**Figure 2:**
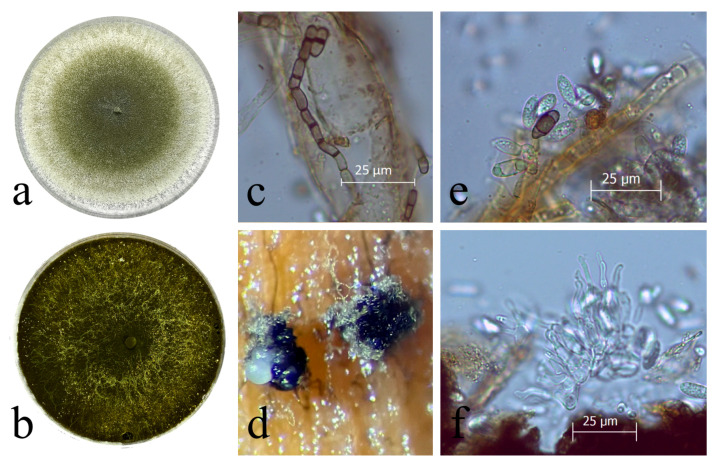
Morphological characteristics of *Neoscytalidium*, 4-day-old colony on PDA (**a**) and 7-day-old colony on PDA (**b**), arthroconidia (**c**), black conidiomata (pycnidia) on wood chips (**d**), conidia formed in pycnidia (**e**), and conidiogenous cells (**f**).

The isolation and cultivation of *Neoscytalidium dimidiatum* are accomplished using conventional methods on diverse media such as Potato Dextrose Agar (PDA), Malt Extract Agar (MEA), and Oatmeal Agar (OA). Initially, colonies appear colorless but undergo a transformation within seven days, transitioning from a pale brown or greenish olivaceous shade to citrine hues at the center (Figure 2a,b). Over time, the colonies become black both on the surface and beneath, accompanied by moderately fluffy mycelium that is suppressed and has smooth edges [2,50,51,52].

*Neoscytalidium dimidiatum*, with an unknown teleomorph, presents two distinct asexual forms known as synanamorphs. The hyphomycetous morphology generates powdery to touch-textured conidia in arthric chains through hyphal fragmentation (Figure 2c). These conidia exhibit diverse shapes—from cylindrical-truncate to oblong-obtuse and doliiform—resembling those seen in the *Scytalidium* genus. They measure 4–16.5 × 2.5–8.5 μm and have thick walls that shift from an initial hyaline state to a dark brown coloration with age [2,50,51,52,53].

The coelomycetous morph generates hyaline, ellipsoidal conidia, which may have zero to two septa and possess a darkened central cell, closely resembling those found in the *Fusicoccum* genus. These conidia measure approximately 10–16 × 3.5–6.5 μm and are enclosed within solitary or multilocular conidiomata, often referred to as pycnidia. These pycnidia can be observed on various substrates, including sterilized pine needles and corn straw, using water agar (Figure 2d,e). The conidiogenous cells responsible for conidia production are holoblastic, cylindrical, and hyaline, with dimensions of 6–14 × 1.5–4 μm (Figure 2f).

For identification purposes, specific gene regions such as ITS, *tef1*, and *tub2* are employed, with *tef1* playing a pivotal role in the accurate identification of *Neoscytalidium* [2,50,51,52,53]. Additionally, the optimum mycelial growth of this fungus occurs at temperatures ranging from 33 to 35 °C, with conidial germination reaching its peak between 38 and 40 °C [11,30,49,72,73,74,75,76].

## 4. Host Range, Symptoms, and Geographic Distribution of *Neoscytalidium dimidiatum*

### 4.1. Host Range and Geographical Distribution Diversity of Neoscytalidium dimidiatum

*Neoscytalidium dimidiatum* demonstrates a wide-ranging infective capacity, encompassing a diverse array of plant species, as substantiated by records that document its presence in 126 distinct host species. Yet, the reliability of these reports remains a challenge due to taxonomic intricacies linked with *N*. *dimidiatum* and its historically rejected synonyms. Throughout history, various checklists have been published as outcomes of extensive surveys of plant pathogens. In recent times, many of these inventories have been amalgamated into comprehensive databases, such as the United States Department of Agriculture (USDA) database. These databases, including the USDA fungal database and similar sources from other countries, stand as invaluable repositories for comprehending the host spectrum and geographic distribution of plant pathogenic fungi. Despite the temporary inactivity of the USDA fungal database, we utilized the list of sources saved in 2018 to illustrate the geographic distribution of *N*. *dimidiatum* and its basionyms and synonyms. To construct an exhaustive dataset, we systematically compiled plant host data from diverse literature sources and the USDA database. This compilation, consisting of 250 instances of *N*. *dimidiatum* and its synonymous taxa from natural habitats, serves as a foundation for analysis (Table 1). This table, meticulously organized, presents a comprehensive listing of hosts associated with *N*. *dimidiatum*. Host species, along with their common names and corresponding families, are alphabetically arranged. Additionally, the table showcases the basionym names of *N*. *dimidiatum* reported in each country, illustrating its global distribution. This well-structured table offers a valuable and up-to-date resource for plant pathologists and researchers delving into the intricate relationship between *N*. *dimidiatum* and its diverse host range across various countries. Leveraging this host information has enabled the validation of *N*. *dimidiatum*’s expansive host range. Presently, the confirmed host count stands at over 100 (126 to date), cementing the understanding of its diverse host interactions. These hosts span 46 distinct families, with 43 of them belonging to the category of seed plants. The list of host families, including Acanthaceae, Anacardiaceae, Apocynaceae, Araliaceae, Berberidaceae, Betulaceae, Boraginaceae, Cactaceae, Casuarinaceae, Combretaceae, Convolvulaceae, Cucurbitaceae, Ebenaceae, Ericaceae, Euphorbiaceae, Fabaceae, Fagaceae, Juglandaceae, Lamiaceae, Lythraceae, Malvaceae, Meliaceae, Moraceae, Myrtaceae, Oleaceae, Proteaceae, Rhamnaceae, Rhizophoraceae, Rosaceae, Rutaceae, Salicaceae, Solanaceae, Ulmaceae, and Vitaceae, collectively fall within the classification of eudicots. These families are categorized under angiosperms, also known as flowering plants. Monocot hosts, represented by the Amaryllidaceae, Araceae, Asparagaceae, Asphodelaceae, Bromeliaceae, Dioscoreaceae, Iridaceae, Musaceae, and Orchidaceae families, constitute a significant subdivision within angiosperms. In particular, within the spectrum of seed plants, the majority of host families are classified as eudicots, making monocot hosts relatively infrequent for *N*. *dimidiatum*. The presence of *N*. *dimidiatum* within Araucariaceae, Cupressaceae, and Pinaceae aligns with the gymnosperms classification. The full host range of this species is not known as, apparently, it can be an endophyte/saprophyte instead of a pathogen in some plant species. Reports lacking the integration of morphological analysis and multigene phylogenetic analysis for precise pathogen identification introduce uncertainty into the existing knowledge. Thus, *N*. *dimidiatum* showcases its ability to infect a broad spectrum of seed plant families and potentially extends to other plant groups, including gymnosperms, establishing itself as one of the most versatile fungal plant pathogens in terms of host range.

The geographic distribution of *N*. *dimidiatum* underscores its global presence, with varying prevalence. The highest number of recorded occurrences, totaling 132, is notably concentrated in Asia, indicating a significant hotspot for this pathogen. North America reports 50 occurrences, suggesting a substantial yet comparatively lower frequency. Africa documents 26 instances, demonstrating the pathogen’s notable presence on the continent. In South America and Australia, 16 and 15 recorded occurrences, respectively, indicate a balanced distribution across these regions. Europe, with 11 reported cases, points to a noteworthy but comparatively lower occurrence rate. In contrast, Oceania reports only a single recorded instance, signifying a rare presence in this region (for all relevant references throughout this chapter, see Table 1).

**Table 1:** Comprehensive compilation of hosts associated with *Neoscytalidium dimidiatum*. The host species, along with their scientific nomenclature and familial categorization, are systematically organized in alphabetical order. Concurrently, the basionym designations of *N. dimidiatum*, as documented within each country, are presented, showcasing its worldwide dispersion.

Plant Species Family	Host Species	Common Host Name	Identified Species Name	Target Loci for IDENTIFICATION	Koch’s Postulates	Symptoms	Country	Continent	References
Acanthaceae	*Avicennia marina*	White mangrove	Nd	ITS	+	Canker and dieback	Iran	Asia	[77]
Amaryllidaceae	*Hymenocallis littoralis*	White spider lily	Nd	ITS	+	Leaf blight	Malaysia	Asia	[78]
	*Clivia miniata*	Natal lily	Nd	ITS, *tef1*	+	Leaf blight	Iran	Asia	[79]
Anacardiaceae	*Anacardium occidentale*	Cashew	Nh	ITS, *tef1*	+	Dieback and stem and branch cankers	Brazil	South America	[72]
	*Mangifera indica*	Mango	Ht	−	+	Leaf spot	India	Asia	[80]
			Ht	−	+	Dieback	Niger	Africa	[81]
			Ef	−	NA	Herbarium specimen records	South Africa	Africa	[57]
			Ht	−	NA	NA	Brazil	South America	[82]
			Fd	ITS, *tub2*	−	NA	US—California	North America	[58]
			Nd and Nn	ITS, *tef1*	+	Dieback	Australia	Australia	[35]
			Nd and Nn	ITS, *tef1*	+	Canker	Australia	Australia	[83]
			Nd	ITS, *tef1*	+	Dieback and stem-end rot	Brazil	South America	[84]
			Nh	ITS, *tef1*	+	Dieback and stem and branch cankers	Brazil	South America	[72]
	*Pistacia vera*	Pistachio	Nd	ITS, *tef1*, *tub2*	+	Endophytic	Iran	Asia	[85]
			Nd	ITS, LSU	+	Canker, shoot blight, and root rot	Turkey	Asia	[42]
			Nn	ITS, *tef1*	+	Dieback	Turkey	Asia	[86]
	*Rhus typhina*	Staghorn sumac	Td	ITS, *tub2*	NA	NA	US—West Virginia	North America	[87,88]
Apocynaceae	*Nerium oleander*	Oleander	Nn	ITS, LSU, *tef1*	−	Sooty canker	Iran	Asia	[40]
Araceae	*Thaumatophyllum bipinnatifidum (Philodendron bipinnatifidum)*	Split-leaf philodendron	Ht	−	NA	NA	India	Asia	[89]
Araliaceae	*Meryta denhamii*	Meryta	Nd	ITS, *tef1*, *tub2*	+	Branch canker and dieback	Italy	Europe	[90]
Araucariaceae	*Agathis robusta (A.palmerstoni)*	Kauri	Ef	−	NA	Herbarium specimen records	Australia	Australia	[57]
	*Araucaria* sp.	Chilean pine	Ht	−	NA	NA	Malaysia	Asia	[91]
Asparagaceae	*Agave* sp.	Century plant	Ht	−	NA	NA	Guinea	Africa	[92]
	*Agave americana*	Century plant	Ef	−	NA	Herbarium specimen records	India	Asia	[57]
	*Agave sisalana*	Sisal	Nd	ITS, *tef1*, *tub2*	+	Black leaf spot	China	Asia	[93]
	*Furcraea foetida (F. gigantea)*	Green aloe	Ht	−	NA	NA	Malaysia	Asia	[94]
	*Sansevieria hyacinthoides (S. guineensis)*	African Bowstring Hemp	Ht	−	NA	NA	Guinea	Africa	[92]
	*Sansevieria trifasciata (Dracaena trifasciata)*	Dracaena	Nd	ITS	+	Leaf blight	Malaysia	Asia	[95]
			Nd	ITS, *tef1*	+	Leaf blight	Brazil	South America	[96]
Asphodeloideae	*Aloidendron dichotomum*	Quiver tree	Nd	ITS, LSU, *tef1*, *tub2*, *chs−1*	−	Epiphyte on stems	South Africa	Africa	[97]
Berberidaceae	*Berberis vulgaris*	Barberry	Nd	ITS, *tef1*, *tub2*, *act*	+	Canker and dieback	Iran	Asia	[98]
Betulaceae	*Alnus glutinosa*	Common alder	Nn	ITS, *tef1*	+	Branch and trunk cankers	Iran	Asia	[1]
	*Carpinus betulus*	Common hornbeam	Nn	ITS, *tef1*	+	Branch and trunk cankers	Iran	Asia	[1]
Boraginaceae	*Cordia myxa*	Assyrian plum	Nn	ITS, LSU, *tef1*	−	Canker	Iran	Asia	[40]
Bromeliaceae	*Ananas comosus (A. sativa)*	Pineapple	Ht	−	NA	Leaf spot; fruit rot	Malaysia	Asia	[94]
			Ht	−	NA	Leaf spot; fruit rot	Tanzania	Africa	[99]
			Ef	−	NA	Herbarium specimen records	Sierra Leone	Africa	[57]
			Ef	−	NA	Herbarium specimen records	Solomon Islands—Rendova	Oceania	[57]
			Nd	ITS, LSU	+	Postharvest stem end rot	Malaysia	Asia	[100]
Cactaceae	*Nopalea cochenillifera*	Pickly pear cactus	Nh	ITS, *tef1*, *tub2*	+	Squamous cladode spots	Brazil	South America	[101]
	*Selenicereus (Hylocereus) undatus*	Pitahaya (white-fleshed dragon fruit)	Nd	ITS	+	Stem canker	Taiwan	Asia	[5]
			Nd	ITS	+	Brown stem-spot-forming canker	China	Asia	[6]
			Nd	ITS, *tub2*	+	Internal black rot	Israel	Asia	[8]
			Nd	ITS	+	Canker, internal brown rot	China	Asia	[7,102]
			Nd	ITS	+	Stem and fruit canker	US—Florida	North America	[10]
			Nd	ITS, *tub2*	+	Stem and fruit canker	US—Florida	North America	[11]
	*Selenicereus (Hylocereus) undatus × S. polyrhizus*	Red-fleshed dragon fruit	Nd	ITS, *tub2*	+	Stem and fruit canker	US—Florida	North America	[11]
	*Selenicereus (Hylocereus) polyrhizus*	Red-fleshed dragon fruit	Nd	ITS	+	Stem canker	Taiwan	Asia	[5]
			Nd	ITS	+	Stem canker	Malaysia	Asia	[9]
			Nd	ITS	+	Stem canker	China	Asia	[12]
			Nd	ITS, LSU, *tub2*	+	Stem canker	Thailand	Asia	[15]
	*Selenicereus (Hylocereus) megalanthus*	Yellow pitahaya (dragon fruit)	Nd	ITS, *tef1*, *tub2*	+	Stem canker	Malaysia	Asia	[17]
			Nd	ITS, *tef1*, *tub2*	+	Stem canker	Ecuador	South America	[18]
	*Selenicereus (Hylocereus) monacanthus*	Dragon fruit	Nd	*tub2*	+	Stem and fruit canker	Philippines	Asia	[13]
	*Selenicereus* spp. (*Hylocereus* spp.)	Dragon fruit	Nd	ITS, *tef1*	+	Stem canker	Puerto Rico	North America	[14]
			Nd	ITS, *tef1*, *tub2*	+	Stem canker	India	Asia	[16]
Casuarinaceae	*Casuarina* sp.	Casuarina	Ht	−	NA	NA	Pakistan	Asia	[103]
Combretaceae	*Conocarpus erectus*	Buttonwood or button mangrove	Nn	ITS, LSU, *tef1*	+	Lamination of the trunk bark	Iran	Asia	[40]
Convolvulaceae	*Ipomoea batatas*	Sweet potato	Ht	−	NA	NA	Malaysia	Asia	[94,104]
			Nd	ITS, *tef1*	+	Root rot	Brazil	South America	[105]
			Nd	ITS, *tef1*	+	Root and stem rot	Brazil	South America	[106]
Cucurbitaceae	*Cucumis melo*	Melon	Nh	ITS	+	Fruit rot	Iran	Asia	[107]
Cupressaceae	*Cupressus sempervirens*	Mediterranean cypress	Nn	ITS, LSU, *tef1*	−	Canker and dieback	Iran	Asia	[40]
	*Sequoiadendron giganteum (Sequoia gigantea)*	Giant redwood, giant sequoia	Ht	−	NA	NA	US—California	North America	[108]
Dioscoreaceae	*Dioscorea esculenta*	Lesser yam	Nd	ITS, *tef1*, *tub2*	+	Dieback	China	Asia	[109]
	*Dioscorea rotundata*	Yam	Nd	ITS, *tub2*	−	Tuber dry rot	Colombia	South America	[110]
Ebenaceae	*Diospyros kaki*	Japanese persimmon	Nn	ITS, *tef1*	+	Branch dieback	Turkey	Asia	[111]
Ericaceae	*Arbutus menziesii*	Madrone	Ht	−	NA	NA	US—Washington	North America	[112,113]
			Ht	−	NA	NA	Canada	North America	[114]
			Ht	−	NA	Canker	US—California	North America	[115]
	*Arbutus unedo*	Strawberry tree	Ht	−	+	Leaf spotting and defoliation	Greece	Europe	[116]
Euphorbiaceae	*Jatropha curcas*	Physic nut, a biofuel plant	Nh	ITS	+	Collar and root rot	Brazil	South America	[117]
			Nd	ITS, *tef1*, *tub2*	+	Collar and root rot	Brazil	South America	[118]
	*Manihot esculenta* (*M. utilissima*)	Cassava	Ht	−	NA	NA	Ghana	Africa	[119]
			Ht	−	NA	NA	Kenya	Africa	[120]
			Ef	−	NA	Herbarium specimen records	Ghana	Africa	[57]
			Nh	ITS, *tef1*, *tub2*	+	Black root rot	Brazil	South America	[121]
			Nd	ITS, *tef1*, *rpb2*	+	Black root rot and stem cutting dry rot	Brazil	South America	[122]
			Nd	ITS, *tef1*	+	Black stem and root rot	Thailand	Asia	[123]
Fabaceae	*Acacia auriculiformis*	Earleaf acacia	Ht	−	NA	NA	India	Asia	[89,124]
			Ef	−	NA	Herbarium specimen records	India	Asia	[57]
	*Acacia melanoxylon*	Australian blackwood	Ht	−	NA	NA	India	Asia	[124,125]
	*Acacia synchronicia*	Bardi bush	Nn	ITS, *tef1*	−	Asymptomatic branches (sapwood)	Australia	Australia	[51]
			Nh	ITS, *tef1*	+	Endophyte as a potential pathogen	Australia	Australia	[126]
	*Albizia lebbeck*	Siris tree	Sd	−	−	Dieback	Oman	Asia	[34]
			Nn	ITS, LSU, *tef1*	−	Sooty canker	Iran	Asia	[40]
	*Bauhinia purpurea*	Orchid tree	Nn	ITS, LSU, *tef1*	+	Lamination of the trunk bark	Iran	Asia	[40]
	*Cassia fistula*	Golden shower tree	Nn	ITS, LSU, *tef1*	−	Canker	Iran	Asia	[40]
	*Cassia floribunda*	Arsenic bush	Nn	ITS, LSU, *tef1*	−	Canker	Iran	Asia	[40]
	*Cicer arietinum*	Chickpea	Nd	ITS, *tef1*, *tub2*	+	Blight and root rot	Turkey	Asia	[49]
	*Crotalaria medicaginea*	Rattlepods	Nn	ITS, *tef1*	−	Asymptomatic branches (sapwood)	Australia	Australia	[51]
			Nd	ITS, *tef1*	+	Endophyte as a potential pathogen	Australia	Australia	[126]
	*Delonix regia*	Royal poinciana	Sd	−	−	Dieback	Oman	Asia	[34]
			Nd	ITS, LSU, *tef1*, *tub2*	+	Stem canker	United Arab Emirates	Asia	[127]
	*Lysiphyllum cunninghamii*	Kimberley bauhinia or jigal tree	Nn	ITS, *tef1*	+	Endophyte as a potential pathogen	Australia	Australia	[126]
	*Parkinsonia aculeata*	Palo verde	Nn	−	−	Dieback (when used as a bipherbicide)	Australia	Australia	[128]
	*Peltophorum petrocarpum*	Copperpod	Sd	−	−	Dieback	Oman	Asia	[34]
Fagaceae	*Castanea sativa*	Sweet chestnut	Ef	−	NA	Canker	US—California	North America	[129]
			Ht	−	NA	Canker	US—California	North America	[115]
	*Fagus orientalis*	Oriental beech	Nn	ITS, *tef1*	+	Branch and trunk cankers	Iran	Asia	[1]
	*Quercus brantii*	Persian oak	Nn	ITS, LSU, *tef1*	+	Dieback	Iran	Asia	[74]
			Nd	ITS, LSU, SSU	+	Decline and sooty canker	Iran	Asia	[130]
Iridaceae	*Gladiolus* sp.	Gladiolus	Ht	−	NA	NA	US—California	North America	[115]
Juglandaceae	*Juglans californica*	California black walnut	Ht	−	NA	On twigs	US—California	North America	[131]
	*Juglans regia*	English walnut	Ef	−	NA	Branch wilt	US—California	North America	[60,129,132]
			Ht	−	NA	NA	US—California	North America	[115,133]
			Fd	ITS, *tub2*	−	NA	US—California	North America	[58,134]
			Nd	ITS, *tef1*, *tub2*	+	Black canker and death of graft union	US—California	North America	[135,136]
			Nd	ITS, LSU, *tef1*	+	Black canker, root rot, decline	Turkey	Asia	[137,138]
			Nd			Decline	Iran	Asia	[139]
			Nn	ITS, LSU, *tef1*	−	Sooty canker	Iran	Asia	[40]
Lamiaceae	*Lavandula angustifolia*	Lavender	Nd	ITS, *tef1*	+	Foliar and stem blight	Turkey	Asia	[140]
	*Melissa officinalis*	Lemon balm	Nd	ITS, *tef1*	+	Blight	Turkey	Asia	[141]
	*Origanum onites*	Turkish oregano	Nd	ITS, *tef1*	+	Leaf blight	Turkey	Asia	[142]
	*Salvia officinalis*	Common sage	Nn	ITS, *tef1*	+	Root rot and foliar blight	Turkey	Asia	[143]
Lythraceae	*Punica granatum*	Pomegranate	Nd	ITS, *tef1*, *tub2*, *act*	+	Necrotic wood tissues	Iran	Asia	[144]
			Nn	ITS, LSU, *tef1*	−	Sooty canker	Iran	Asia	[40]
Malvaceae	*Adansonia gibbosa*	Baobab	Nn	ITS, *tef1*	−	Asymptomatic branches (sapwood)	Australia	Australia	[51]
	*Adansonia gregorii*	Boab	Nh	ITS, *tef1*	+	Endophyte as a potential pathogen, canker	Australia	Australia	[126]
	*Hibiscus rosa-sinensis*	Chinese hibiscus	Nn	ITS, LSU, *tef1*	−	Canker and dieback	Iran	Asia	[40]
	*Thespesia populnea*	Portia tree	Sd	−	−	Dieback	Oman	Asia	[34]
Meliaceae	*Azadirachta indica*	Neem	Nh	ITS, *tef1*, *tub2*, *act*	+	Decline	Iran	Asia	[145]
	*Melia azedarach*	Chinaberry	Ht	−	−	NA	Pakistan	Asia	[146]
			Nd	ITS, LSU, *tub2*	−	Canker and dieback	Iraq	Asia	[147]
Moraceae	*Ficus benghalensis*	Indian banyan	Sd	−	−	Dieback	Oman	Asia	[34]
			Nd			Sooty canker	Iran	Asia	[39]
			Nn	ITS, LSU, *tef1*	−	Dieback and sooty canker	Iran	Asia	[40]
	*Ficus benjamina*	Weeping fig	Nd	ITS, LSU, *rpb2*		Branch dieback	Mexico	North America	[36]
			Nd	ITS, LSU, SSU	+	Sooty canker	Egypt	Africa	[37]
	*Ficus carica*	Common fig	Ht	−	−	NA	Cyprus	Europe	[148]
			Ht	−	NA	NA	US—California	North America	[115,131,149]
			Ht	−	NA	Herbarium specimen records	Cyprus	Europe	[57]
			Sd	−	−	Dieback	Oman	Asia	[34]
			Nd	ITS, *tef1*	+	Dieback	Australia	Australia	[35]
			Nd	ITS, *tef1*, *tub2*	+	Dieback and canker	Turkey	Asia	[38]
			Nd	−		Limb dieback	US—California	North America	[41]
	*Ficus nitida*	Chinese banyan	Nd	ITS, LSU, SSU	+	Sooty canker	Egypt	Africa	[37]
	*Ficus religiosa*	Bodhi tree	Nn	ITS, LSU, *tef1*	+	Dieback and sooty canker	Iran	Asia	[40]
	*Ficus retusa*	Banyan tree	Sd	−	−	Dieback	Oman	Asia	[34]
	*Morus alba*	White mulberry	Ef	−	NA	Herbarium specimen records	Pakistan	Asia	[57]
			Ef	−	NA	Herbarium specimen records	US	North America	[57]
			Ht	−	−	NA	Pakistan	Asia	[146]
			Nn	ITS, LSU, *tef1*	+	Shoot and branch deaths	Turkey	Asia	[150]
	*Morus bombycis* (*Morus australis*)	Korean mulberry	Nn	ITS, LSU, *tef1*	+	Branch necrosis	Turkey	Asia	[150]
	*Morus nigra*	Black mulberry	Nn	ITS, LSU, *tef1*	+	Shoot and branch deaths	Turkey	Asia	[150]
			Nn	ITS, LSU, *tef1*	+	Sooty canker	Iran	Asia	[40]
Musaceae	*Musa nana*	Banana (dwarf)	Ht	−	−	Tip rot	Jamaica	North America	[151]
	*Musa* spp.	Banana	Ht	−	−	Tip rot	Hawaii	North America	[88,152]
	*Musa acuminata*	Banana	Ht	−	−	Leaf spot	Hawaii	North America	[153]
Myrtaceae	*Callistemon viminalis*	Weeping bottlebrush	Nn	ITS, LSU, *tef1*	−	Canker	Iran	Asia	[40]
	*Eucalyptus camaldulensis*	River red gum	Nd	ITS	+	Sooty canker	Iraq	Asia	[154]
			Nn	ITS, LSU, *tef1*	+	Dieback, lamination of the trunk bark, and sooty canker	Iran	Asia	[40]
	*Eucalyptus* sp.	Eucalyptus	Ef	−	NA	Herbarium specimen records	Portugal	Europe	[57]
			Nn	ITS, *tef1*	+	Endophyte as a potential pathogen	Australia	Australia	[126]
	*Eucalyptus* spp.	Eucalyptus	Nd	ITS, LSU, *tub2*	−	Canker and dieback	Iraq	Asia	[147]
	*Psidium guajava*	Guava	Ht	−	NA	NA	India	Asia	[104]
			Nh	ITS, *tef1*	+	Dieback and stem and branch cankers	Brazil	South America	[72]
			Nd	ITS, *tef1*	+	Postharvest fruit rot	Malaysia	Asia	[155]
	*Syzygium cumini*	Java plum	Nh	ITS, *tef1*, *tub2*, *act*	+	Cankers and wedge-shaped wood necrosis	Iran	Asia	[156]
			Nh	ITS, *tef1*, *tub2*, *act*	+	Asymptomatic wood tissue	Iran	Asia	[156]
			Nn	ITS, LSU, *tef1*	+	Lamination of the trunk bark	Iran	Asia	[40]
Oleaceae	*Olea europaea*	Olive	Nd	ITS, *tef1*, *tub2*	+	Canker and leaf scorch	Turkey	Asia	[76]
Orchidaceae	*Arachnis* sp.	Scorpion orchid	Ht	−	−	NA	Malaysia	Asia	[157]
	*NA*	Orchid	No	ITS, LSU	−	From a fallen orchid leaf	Thailand	Asia	[53]
	*Cattleya lueddemanniana* var. *lueddemanniana*	Cattleya orchid	No	ITS, LSU	+	Leaf spot	Thailand	Asia	[158]
	Cattleya × hybrid	Orchids	Nd	ITS	+	Leaf blight	Taiwan	Asia	[159]
Pinaceae	*Picea pungens*	Blue spruce	Nd	ITS, *tef1*, *tub2*	+	Needle blight	Turkey	Asia	[160]
	*Pinus eldarica*	Afghan pine	Nd	ITS, LSU	+	Shoot and needle blight	Turkey	Asia	[27]
			Nn	ITS, LSU, *tef1*	+	Dieback	Iran	Asia	[28]
	*Pinus nigra*	European black pine	Nd	ITS, LSU	+	Shoot and needle blight	Turkey	Asia	[27]
	*Pinus sylvestris*	Scots pine	Nd	ITS, LSU	+	Shoot and needle blight	Turkey	Asia	[27]
Proteaceae	*Grevillia agrifolia*	Blue grevillea	Nn	ITS, *tef1*	−	Asymptomatic branches (sapwood)	Australia	Australia	[51]
			Nn	ITS, *tef1*	+	Endophyte as a potential pathogen	Australia	Australia	[126]
Rhamnaceae	*Ziziphus spina-christi*	Christ’s thorn jujube	Nn	ITS, LSU, *tef1*	−	Canker	Iran	Asia	[40]
Rhizophoraceae	*Rhizophora mucronata*	Red mangrove	Nd	ITS	+	Canker and dieback	Iran	Asia	[77]
Rosaceae	*Malus domestica (M. pumila)*	Apple	Ht	−	NA	Gummosis and dieback	Egypt	Africa	[56]
			Ht	−	NA	NA	India	Asia	[89]
			Ef	−	NA	Herbarium specimen records	India	Asia	[57]
			Ef	−	NA	Herbarium specimen records	Iraq	Asia	[57]
			Nd			Branch canker	Iran	Asia	[161]
			Nd	ITS, *tef1*	+	Branch dieback and canker	Turkey	Asia	[32]
			Nd			Cankers	China	Asia	[162]
	*Prunus armeniaca*	Apricot	Ht	−	NA	Gummosis and dieback	Egypt	Africa	[56]
			Ht	−	−	NA	Cyprus	Europe	[148]
			Ht	−	NA	Herbarium specimen records	Cyprus	Europe	[57]
			Ht	−	NA	Canker	US—California	North America	[115,131]
			Nd	ITS, LSU, *tef1*, *tub2*	+	Shoot blight, branch dieback, and canker	Turkey	Asia	[31]
	*Prunus avium*	Cherry	Nn	ITS, *tef1*	+	Canker and branch dieback	Turkey	Asia	[163]
	*Prunus domestica*	Plum	Ht	−	NA	Gummosis and dieback	Egypt	Africa	[56]
			Nd	ITS	+	Decline and dieback	Tunisia	Africa	[29]
			Nn	ITS, *tef1*	+	Branch dieback and stem cankers	Turkey	Asia	[164]
	*Prunus dulcis* (*P. amygdalus*)	Almond	Ht	−	+	Secondary canker infection	US—California	North America	[165]
			Ht	−	NA	Canker	US—California	North America	[115,131]
			Nd	ITS, *tef1*, *tub2*	+	Trunk and branch cankers, spur and shoot blight, fruit rot	US—California	North America	[30]
			Nn	ITS, *tef1*	+	Stem canker and branch dieback	Turkey	Asia	[32]
			Nd	ITS, *tef1*, *tub2*, *GPD*	+	Trunk and branch cankers	US—California	North America	[33]
	*Prunus persica*	Peach	Ht	−	−	Canker	US—California	North America	[115,131]
			Nn	ITS, LSU, *tef1*	−	Sooty canker	Iran	Asia	[40]
	*Prunus* sp.	Prunus	Fd	ITS, *tef1*, *tub2*	+	NA	Egypt	Africa	[58,134,166]
	*Pyrus communis*	Pear	Nn	ITS, LSU, *tef1*	+	Shoot blight and branch canker	Turkey	Asia	[167]
Rutaceae	*Citrus aurantifolia*	Acid lime	Ht	−	NA	NA	US—California	North America	[115,131]
			Nd	ITS	+	Root rot	Oman	Asia	[46]
			Nh	ITS, *tef1*, *tub2*	+	Canker and dieback	Iran	Asia	[168]
			Nn	ITS, LSU, *tef1*	−	Canker and dieback	Iran	Asia	[40]
	*Citrus clementina*	Clementine	Nd	ITS, *tub2*	+	Shoot blight	Jordan	Asia	[169]
	*Citrus latifolia*	Persian lime	Ht	−	NA	NA	US—California	North America	[115,131]
	*Citrus limetta*	Sweet limetta	Nh	ITS, *tef1*, *tub2*	+	Canker and dieback	Iran	Asia	[168]
			Nn	ITS, LSU, *tef1*	−	Dieback	Iran	Asia	[40]
	*Citrus limettioides*	Sweet lime	Nd	ITS	+	Root rot	Oman	Asia	[46]
	*Citrus limon* (*C. limonium*)	Lemon	Ht	−	−	NA	Cyprus	Europe	[148]
			Ef	−	NA	NA	US—California	North America	[129]
			Ht	−	NA	NA	US—California	North America	[115,131]
			Nh	ITS, *tef1*, *tub2*	+	Branch canker	US—California	North America	[20]
	*Citrus maxima* (*C. grandis*)	Pomelo	Ht	−	NA	NA	US—California	North America	[131]
			Ht	−	NA	NA	US—California	North America	[115]
			Nd	ITS, *tub2*	+	Shoot blight	Jordan	Asia	[169]
	*Citrus meyerii*	Meyer lemon	Ht	−	NA	NA	US—California	North America	[131]
	*Citrus paradisi*	Grapefruit	Ht	−	NA	Canker, dieback	US—California	North America	[115,131,170]
			Ef	−	NA	NA	US—California	North America	[129]
			Nd	ITS, *tef1*, *tub2*	+	Bot gummosis	US—California	North America	[171]
			Nh	ITS, *tef1*, *tub2*	+	Branch canker	US—California	North America	[20]
			Nd	ITS, *tub2*	+	Shoot blight	Jordan	Asia	[169]
	*Citrus reticulata*	Mandarin	Ht	−	NA	NA	US—California	North America	[115,131]
	*Citrus sinensis*	Sweet orange	Ht	−	NA	NA	South Africa	Africa	[172]
			Ht	−	NA	NA	US—California	North America	[115,131]
			Ef	−	NA	Herbarium specimen records	Pakistan	Asia	[57]
			Nd	ITS	+	Blight, canker, and gummosis	Italy	Europe	[19]
			Nn	ITS, LSU, *tef1*	−	Dieback	Iran	Asia	[40]
	*Citrus* sp.	Citrus	Td	−	NA	NA	US—California	North America	[129]
			Ht	−	NA	NA	US—California	North America	[115,129]
	*Citrus tangelo*	Tangelo	Ht	−	NA	NA	US—California	North America	[115,131]
Salicaceae	*Populus alba*	Silver poplar	Ht	−	−	NA	Cyprus	Europe	[148]
			Ht	−	NA	Herbarium specimen records	Cyprus	Europe	[57]
	*Populus fremontii*	Frémont’s cottonwood	Ht	−	NA	NA	US—California	North America	[173]
	*Populus nigra*	Black poplar	Nh	*tef1*	+	Decline	Iran	Asia	[43]
	*Salix alba*	White willow	Nh	*tef1*	+	Decline, irregular and central wood necrosis	Iran	Asia	[43]
			Nd	ITS, LSU	+	Dieback, shoot blight, and branch canker	Turkey	Asia	[44]
Solanaceae	*Capsicum annuum*	Pepper	Ht	−	NA	NA	Tanzania	Africa	[99]
	*Solanum lycopersicum*	Tomato	Nd	ITS, LSU, *tef1*	+	Blight and root rot	Turkey	Asia	[47]
		Tomato	Nn	ITS, LSU, *tef1*	+	Stem blight	Turkey	Asia	[174]
	*Solanum tuberosum*	Potato	Nd	ITS, LSU, *tef1*	+	Tuber rot	Turkey	Asia	[175]
Ulmaceae	*Ulmus* sp.	Elm	Nh	ITS, *tef1*	+	Decline	Iran	Asia	[176]
Vitaceae	*Vitis vinifera*	Grapevine	Ht	−	NA	Drying	India	Asia	[89,177,178]
			Ht	−	NA	Branch wilt	Iraq	Asia	[179]
			Nd	ITS	+	Dieback	Iraq	Asia	[21]
			Nd	ITS, *tub2*	+	Wood canker and decline	US—California	North America	[22]
			Nh	ITS, *tef1*	+	Dieback	Brazil	South America	[23]
			Nd	ITS, LSU, *tef1*, *tub2*	+	Canker and dieback	Turkey	Asia	[24]
			Nn	ITS, *tef1*	+	Wood canker	Turkey	Asia	[25]
			Nh	ITS	+	Wood necrosis	Iran	Asia	[26]

In the ‘Identified species name’ column: Td for *Torula dimidiata*, Ht for *Hendersonula toruloidea*, Ef for *Exosporina fawcettii*, Sh for *Scytalidium hyalinum*, Sd for *Scytalidium dimidiatum*, Fd for *Fusicoccum dimidiatum*, Nd for *Neoscytalidium dimidiatum*, Nn for *Neoscytalidium novaehollandiae*, Nh for *Neoscytalidium hyalinum*, and No for *Neoscytalidium orchidacearum*. In the ‘Target loci for identification’ column: ITS: Internal Transcribed Spacer region, *tef1*: Translation Elongation Factor 1-alpha gene, *tub2*: Beta-tubulin gene, LSU: large subunit (of ribosomal RNA) gene, SSU: small subunit (of ribosomal RNA) gene, *chs-1*: chitin synthase 1, *act*: Actin gene, rpb2: RNA Polymerase II Second Largest Subunit gene, and *GPD*: Glyceraldehyde-3-Phosphate Dehydrogenase gene. In the ‘Koch’s Postulates’ column, ‘−’ indicates unfulfilled Koch’s postulates, signifying a lack of demonstrated pathogenicity; ‘+’ indicates fulfilled Koch’s postulates, confirming demonstrated pathogenicity. In the context of this dataset, ‘NA’ indicates that the information is not available.

The distribution of *N*. *dimidiatum* extends across 37 countries, highlighting its adaptability and prevalence across diverse geographic regions. Iran records the highest number of occurrences, with 47 documented instances, emphasizing a significant presence in the region. Turkey closely follows with 30 reported occurrences, highlighting its substantial distribution. Brazil reports 14 instances, underlining the pathogen’s presence in South America. In Asia, Malaysia and China contributed 12 and 6 reports, respectively, indicating a significant distribution. The United States, particularly in California, displays a substantial presence, with 38 occurrences. Various other regions, including Canada, Colombia, Ecuador, Greece, Israel, Italy, Jamaica, Kenya, Mexico, Niger, the Philippines, Portugal, Puerto Rico, Sierra Leone, Solomon Islands—Rendova, Tanzania, Tunisia, the United Arab Emirates, US—Florida, US—Washington, and US—West Virginia, each report a limited number of cases. Notably, countries in the Middle East, such as Iraq and Oman, document six and nine occurrences, respectively, indicating a significant regional presence. This comprehensive distribution analysis underscores the global adaptability of *N*. *dimidiatum* and highlights the need for further research to understand the factors influencing its distribution and to develop effective management strategies.

### 4.2. Diversity of Host Responses and Geographic Distribution Patterns of Neoscytalidium dimidiatum across Plant Families and Countries

*Neoscytalidium dimidiatum* has the ability to infect various parts of its host plants, resulting in a wide range of symptoms, including decline, diebacks of branches and limbs, branch wilting, cankers on stems and branches, sooty cankers, wood cankers leading to necrosis, blights affecting spurs and shoots, needle blights, leaf blights, leaf spots, leaf scorching, blights affecting all above-ground plant parts, gummosis, loss of graft union viability, root rot, black root rot, dry rot in stem cuttings, collar rot, stem end rots after harvest, stem rots, fruit rots, internal brown or black stem and fruit rots, tuber rots, lamination of trunk bark, tip rot, as well as instances of asymptomatic conditions. However, the symptoms induced by the pathogen may resemble those caused by other biotic agents (fungi, bacteria, etc.) or abiotic factors. Identification of *N*. *dimidiatum* solely through visual examination of its host plants is unlikely, as the fruiting structures of the pathogen’s *Fusicoccum*-like (pycnidia with conidia) and/or *Scytalidium*-like synanamorphs (arthrocondia or phragmospores) found on symptomatic plant tissues exhibit morphological similarities to those of other fungal species within the family Botryosphaeriaceae. Moreover, the pathogen can exist in a quiescent or latent state within asymptomatic hosts. Consequently, alternative diagnostic methods beyond visual inspection are necessary for the accurate detection of *N*. *dimidiatum*. However, we include all synonym and basionym names of the pathogen to search the literature for the below host range since there is no similar extensive report to support our understanding of host diversity. In order to comprehensively understand the diversity of hosts, we included all synonymous and basionym names of the pathogen while performing literature searches for the host range. This is necessary as there is a lack of thorough reports on this topic. In recent publications, all identified species have undergone phylogenetic analyses based on DNA datasets.

Cankers and the development of internal brown or black rot caused by *N*. *dimidiatum* represent significant concerns affecting the stems (cladodes) and fruits of dragon fruits (pitahayas) within the *Selenicereus* genus (including *S*. *megalanthus*, *S*. *monacanthus*, *S*. *polyrhizus*, and *S*. *undatus*) belonging to the Cactaceae family. This phenomenon has been observed across various regions, encompassing China, India, Israel, Malaysia, the Philippines, Taiwan, Thailand, Florida, Puerto Rico, and Ecuador. Plants affected by *N*. *dimidiatum* canker exhibit poor recovery as the initial canker spots progress to stem rot, as reported by Chuang et al. [5]. The presence of *N*. *dimidiatum* in fruits leads to decay, resulting in unsatisfactory fruit pulp quality, according to Ezra et al. [8]. These collective studies contribute to a comprehensive understanding of the challenges posed by cankers and internal rot in dragon fruit cultivation. These challenges have garnered significant attention due to their influence on the overall health and vitality of these dragon fruit species. Feijo et al. [101] also identified *N*. *dimidiatum* as the cause of squamous cladode spots on *Nopalea cochenillifera* cactus (Cactaceae), representing the first worldwide record of its presence on this host (for all relevant references throughout this chapter, see Table 1).

*Neoscytalidium dimidiatum* causes canker-related symptoms on a variety of *Ficus* species belonging to the Moraceae family, including *F*. *benghalensis*, *F*. *benjamina*, *F*. *carica*, *F*. *nitida*, *F*. *religiosa*, and *F*. *retusa*. When cankers appear, the overall health of the plant declines rapidly, as evidenced by the death of leaves and branches. Sooty cankers are observed to form on banyans (*F*. *benghalensis* and *F*. *nitida*), bodhi trees (*F*. *religiosa*), and weeping fig (*F*. *benjamina*). These cankers can be distinguished by their dark and encrusted external appearance [37,39,40]). Bark necrosis, the presence of cankers on branches and aerial roots, yellowing of foliage, defoliation, and branch dieback are further signs that have been identified in these plants. In the case of common figs (*F*. *carica*), it is observed that the occurrence of cankers on branches is a frequent phenomenon, leading to the manifestation of dieback symptoms and a subsequent reduction in the overall health of fig trees. These symptoms, if left unaddressed, can ultimately result in the demise of the affected trees. *Neoscytalidium dimidiatum* on *Ficus* spp. exhibits a broad geographical zone of influence, encompassing many regions, including Egypt, Oman, Iran, Turkey, Brazil, California, and Mexico. *Neoscytalidium dimidiatum* also leads to shoot and branch deaths as well as sooty canker in *Morus* spp. (*M*. *alba*, *M*. *australis*, and *M*. *nigra*) of the same family (Moraceae), spanning across regions including Iran, Pakistan, Turkey, and the US.

Symptoms attributed to *N*. *dimidiatum* across diverse citrus hosts encompass a spectrum of manifestations, including canker formation, dieback, shoot blight, branch canker development, bot gummosis, gummosis occurrences, as well as root rot, affecting acid lime (*C*. *aurantifolia*), clementine (*C*. *clementine*), sweet limetta (*C*. *limetta*), sweet lime (*C*. *limettioides*), lemons (*C*. *limon*), pomelo (*C*. *maxima*), grapefruit (*C*. *paradise*), and sweet orange (*C*. *sinensis*) within the *Citrus* spp. domain of the Rutaceae family. These symptoms of *Citrus* spp. have been documented across various geographic regions, including Oman, Iran, Jordan, Pakistan, California, Cyprus, Italy, and Southern Africa.

Symptoms associated with *N*. *dimidiatum* on apple trees (*Malus domestica*), a member of the Rosaceae family, encompass branch dieback, branch canker, and gummosis [180]. These manifestations have been observed within distinct geographical locales, namely Egypt, Iran, India, Iraq, China, and Turkey. Within the sphere of stone fruit trees (*Prunus* spp.) in the Rosaceae family, a manifold spectrum of pathological manifestations becomes apparent. These include spur and shoot blights, branch dieback, trunk and branch cankers, decline, sooty canker, as well as secondary canker infections. Notably, this intricate array of symptoms is observed in apricots (*P*. *armeniaca*), cherries (*P*. *avium*), almonds (*P*. *amygdalus*), and plums (*P*. *dulcis*). These observations have been exhaustively documented across a geographically extensive scope spanning Egypt, Tunisia, Turkey, Iran, California, and Cyprus. The presence of *N*. *dimidiatum* on pear trees (*Pyrus communis*) within the Rosaceae family has been documented in Turkey, where it has been found to be responsible for the development of shoot blight and branch canker.

*Neoscytalidium dimidiatum*’s influence on walnuts (*Juglans* spp.), encompassing both *J*. *regia* and *J*. *californica* of the Juglandaceae family, includes black canker, root rot, graft union failure resulting in death, overall decline, sooty canker formation, as well as instances of branch wilt. These symptoms have been documented within geographical regions spanning Iran, Turkey, and California.

Within the Pinaceae family, *N*. *dimidiatum* has been associated with the occurrence of shoot and needle blight, concomitant with dieback, across diverse species of pine trees (*Pinus* spp.), including *P*. *eldarica*, *P*. *nigra*, and *P*. *sylvestris*. These occurrences have been observed in both Turkish and Iranian settings. The phenomenon of needle blight has been documented in blue spruce (*Picea pungens*) in Turkey.

Members of the Anacardiaceae family, namely mango (*Mangifera indica*) and pistachio (*Pistacia vera*), exhibit susceptibility to the influence of *N*. *dimidiatum*. Mango trees manifest a diverse spectrum of symptoms, encompassing dieback, the formation of stem and branch cankers, as well as the occurrence of leaf spots, evident across extensive geographic regions spanning Brazil, South Africa, Niger, Australia, California, and India. Similarly, pistachio trees within the territories of Turkey and Iran display a varied range of conditions, encompassing canker, shoot blight, and root rot, as well as instances of asymptomatic occurrences.

*Neoscytalidium dimidiatum* induces leaf blight in the white spider lily (*Hymenocallis littoralis*) and natal lily (*Clivia miniata*) of the Amaryllidaceae family and dracaena (*Dracaena trifasciata*) of the Asparagaceae family, with occurrences documented in Malaysia and Iran. Additionally, this fungus is associated with black leaf spots on sisal plants (*Agave sisalana*) of the Asparagaceae family, located in China. Notably, *N*. *dimidiatum* also prompts leaf blight and leaf spot on Cattleya orchids (Cattleya × hybrid, *C*. *lueddemanniana* var. *lueddemanniana*) within the Orchidaceae family in Taiwan and Thailand. Moreover, *N*. *dimidiatum* triggers leaf scorch in olive trees (*Olea europaea*) of the Oleaceae family in Turkey. This decline initiates with initial foliar scorching, which progresses to dieback in twigs, branches, and even entire trees. As the disease advances, necrosis becomes evident, accompanied by the formation of cankers on trunks, branches, and twigs. *N*. *dimidiatum* induces tip rot and leaf spot in bananas (*Musa* spp.: *M*. *acuminata* and *M*. *nana*) of the Musaceae family, noted in Hawaii and Jamaica but solely recorded in checklists. Additionally, foliar and stem blight, along with root rot, affect tomatoes (*Solanum lycopersicum*), and tuber rot impacts potatoes (*S. tuberosum*), both belonging to the Solanaceae family in Turkey.

Postharvest stem end rot and leaf spot in pineapple (*Ananas comosus*) within the Bromeliaceae family in Malaysia are among the symptoms of *N*. *dimidiatum*. Both Brazil and Malaysia have the challenge of dealing with root and stem rots that impact sweet potatoes (*Ipomoea batatas*) from the Convolvulaceae family. Fruit rot is observed in melons (*Cucumis melo*) belonging to the Cucurbitaceae family, with a particular occurrence in Iran. Yams belonging to the Dioscoreaceae family, such as *Dioscorea esculenta* and *Dioscorea rotundata*, experience dieback and tuber dry rot in Iran and Colombia caused by *N*. *dimidiatum*. In Turkey, it has been shown that Japanese persimmons (*Diospyros kaki*) belonging to the Ebenaceae family are susceptible to branch dieback caused by *N*. *dimidiatum*. Within the Euphorbiaceae family, *N*. *dimidiatum* has been observed to cause collar and root rot in the physic nut plant (*Jatropha curcas*) in Brazil. Cassava (*Manihot esculenta*), a member of the Euphorbiaceae family, is confronted with the challenges posed by black root rot and stem-cutting dry rot, which have been observed in several regions, such as Thailand, Kenya, Ghana, and Brazil. In Iran, barberry (*Berberis vulgaris*) shrubs in the Berberidaceae family exhibit symptoms of canker and severe dieback due to *N*. *dimidiatum*.

There are a significant number of studies that delve into the host diversity of *N*. *dimidiatum* within the Fabaceae family. The impact of this pathogen within the family is exemplified by the manifestation of dieback and sooty canker on the siris tree (*Albizia lebbeck*), observed in regions including Iran and Oman. *Neoscytalidium dimidiatum*’s effect extends to the orchid tree (*Bauhinia purpurea*) bark in India, where infection leads to the occurrence of lamination. Canker is a notable affliction affecting both the golden shower tree and arsenic bush (*Cassia* spp., particularly *C*. *fistula* and *C*. *floribunda*) in Iran. Chickpea plants (*Cicer arietinum*) in Turkey are susceptible to *N*. *dimidiatum* infection, resulting in blight and root rot. Dieback and stem canker are prevalent issues observed in the United Arab Emirates and Oman concerning the royal poinciana (*Delonix regia*). Similarly, documented instances of dieback occur in copperpod trees (*Peltophorum petrocarpum*) in Oman. The phenomenon of dieback also extends to *Parkinsonia aculeata*, commonly known as Palo verde trees, located in Australia. This occurrence follows the application of *N*. *dimidiatum* as a bioherbicide. Nevertheless, we firmly advocate against the utilization of this potentially hazardous pathogen as a herbicide.

In the context of the Fagaceae family, investigations into the host diversity of *N*. *dimidiatum* reveal distinct findings. Notable occurrences encompass branch and trunk cankers observed on oriental beech (*Fagus orientalis*) in Iran. Persian oak (*Quercus brantii*) in Iran demonstrates a complex presentation, including dieback, decline, and sooty canker. Moreover, canker affects sweet chestnut (*Castanea sativa*); however, its documentation is solely based on checklist inclusion.

Within the Lamiaceae family, *N*. *dimidiatum* exhibits diverse manifestations. Lavender (*Lavandula angustifolia*) in Turkey encounters foliar and stem blight. Lemon balm (*Melissa officinalis*) experiences blight, while Turkish oregano (*Origanum onites*) suffers from leaf blight in the same region. Common sage (*Salvia officinalis*) faces the combined threat of root rot and foliar blight in the Lamiaceae family context in Turkey.

Within the Myrtaceae family, *N*. *dimidiatum* exhibits a spectrum of host responses, including dieback, trunk bark lamination, canker, sooty canker, and asymptomatic occurrences within *Eucalyptus* species (*E*. *camaldulensis* and other unidentified species) in regions spanning Iran, Iraq, Portugal, and Australia. Guava (*Psidium guajava*) shows postharvest fruit rot, dieback, and stem and branch cankers across Malaysia, India, and Brazil. Similarly, Java plum (*Syzygium cumini*) manifests trunk bark lamination, cankers, and wedge-shaped wood necrosis alongside latent conditions in Iran. Furthermore, *N*. *dimidiatum* induces canker in the weeping bottlebrush (*Callistemon viminalis*) in Iran. This array of symptoms underscores the intricate influence of *N*. *dimidiatum* within the Myrtaceae family.

Within the Salicaceae family, *N*. *dimidiatum* induces decline in poplar trees (*Populus* spp.: *P*. *alba*, *P*. *fremontii*, and *P*. *nigra*), evident across regions including Iran, California, and Cyprus. Moreover, this pathogen is associated with a spectrum of symptoms in willow trees (*Salix alba*) within the same family, encompassing decline, irregular and central wood necrosis, dieback, shoot blight, and branch canker, primarily observed in Iran and Turkey.

*Neoscytalidium dimidiatum*’s impact is diverse across various plant species, resulting in a range of symptoms. For instance, it leads to elm decline (*Ulmus* sp.) within the Ulmaceae family in Iran. In the Vitaceae family, grapevine (*Vitis vinifera*) experiences canker, dieback, wood canker, necrosis, decline, and branch wilt, spreading through regions like Iran, Iraq, Turkey, India, California, and Brazil. Similarly, in the Rhizophoraceae family, red mangrove (*Rhizophora mucronata*) suffers from canker and dieback in Iran. *N*. *dimidiatum* triggers sooty canker and necrotic wood tissues in pomegranate (*Punica granatum*) of the Lythraceae family in Iran, canker and dieback in chinaberry (*Melia azedarach*) of the Meliaceae family in Iraq and Pakistan, and decline in neem (*Azadirachta indica*) of the Meliaceae family in Iran. Additionally, *N*. *dimidiatum* causes lamination of trunk bark in buttonwood (*Conocarpus erectus*) within the Combretaceae family in Iran. Moreover, cankers and dieback affect white mangrove (*Avicennia marina*) in the Acanthaceae family, evident in Iran, as well as dieback of the portia tree (*Thespesia populnea*) of the Malvaceae family in Oman. *N*. *dimidiatum* also induces branch and trunk cankers in both common alder (*Alnus glutinosa*) and common hornbeam (*Carpinus betulus*) of the Betulaceae family in Iran. Certain reports predominantly concentrate on the isolation and identification of field symptoms, while the investigation into pathogenicity aspects remains largely unexplored. In this context, *N*. *dimidiatum* was isolated from canker-infected plants, namely Assyrian plum (*Cordia myxa*) of the Boraginaceae family, oleander (*Nerium oleander*) of the Apocynaceae family, Christ’s thorn jujube (*Ziziphus spina-christi*) within the Rhamnaceae family, Mediterranean cypress (*Cupressus sempervirens*) of the Cupressaceae family, and Chinese hibiscus (*Hibiscus rosa-sinensis*) of the Malvaceae family, all originating from Iran. In Italy, there has been a documented case of branch canker and dieback in meryta (*Meryta denhamii*) (Araliaceae) attributed to *N*. *dimidiatum*. This diverse array of symptoms underscores the fungus’s varied impact.

Remarkably, some hosts exhibit an intriguing phenomenon of asymptomatic conditions, underscoring a distinctive interaction between the pathogen and the host. Notably, these conditions deviate from the conventional endophytic or epiphytic manifestations. Within this context, a noteworthy observation pertains to six genera that serve as symptomless hosts. This exclusive group encompasses acacias (*Acacia* spp.) (Fabaceae), rattlepods (*Crotalaria medicaginea*) (Fabaceae), Kimberley bauhinia (*Lysiphyllum cunninghamii*) (Fabaceae), baobabs (*Adansonia* spp.) (Malvaceae), and blue grevillea (*Grevillea agrifolia*) (Proteaceae) in Australia, alongside quiver tree (*Aloidendron dichotomum*) (Asphodelaceae) in Africa. Given the intricate dynamics and uncertainties surrounding the pathogen’s role, we have chosen to identify these hosts as symptomless, as the exact nature of their interaction with *N*. *dimidiatum* remains to be elucidated.

In summary, *N*. *dimidiatum* demonstrates extensive host diversity, involving 84 distinct plant genera and 126 species distributed across 46 families, which encompass 34 eudicot angiosperms, 9 monocot angiosperms, and 3 gymnosperms. It is noteworthy that among these genera, 10 (*Rhus*, *Thaumatophyllum*, *Agathis*, *Araucaria*, *Furcraea*, *Casuarina*, *Sequoiadendron*, *Gladiolus*, *Arachnis*, and *Capsicum*) lack descriptions regarding the induction of symptoms. Additionally, six genera have been identified as hosting the pathogen in an epiphytic or endophytic capacity without resulting in discernible symptoms. Conversely, detailed investigations have established the symptomatic relationship between *N*. *dimidiatum* and 68 genera, providing comprehensive insights into this aspect. Furthermore, 18 species (*Rhus typhina*, *Thaumatophyllum bipinnatifidum*, *Agathis robusta*, *Araucaria* sp., *Agave* sp., *Furcraea foetida*, *Sansevieria hyacinthoides*, *Casuarina* sp., *Sequoiadendron giganteum*, *Gladiolus* sp., *Arachnis* sp., *Citrus latifolia*, *Citrus meyerii*, *Citrus reticulata*, *Citrus tangelo*, *Populus alba*, *Populus fremontii*, and *Capsicum annuum*) have been recognized as potential hosts, primarily based on checklist inclusion, although specific symptom definitions remain unavailable for these species.

While *Neoscytalidium* species are commonly known as phytopathogens, they can manifest in various clinical conditions, affecting individuals with underlying factors or even those without apparent health issues. Despite typically causing superficial skin and nail infections, these species have been linked to more severe conditions like cerebral phaeohyphomycosis [4]. Notably, Heidari et al. [3] explored *N*. *dimidiatum*’s presence in human respiratory tracts, highlighting its adaptability to different environments and raising important questions about its impact on both human and plant health. This underscores the need for further research into the potential risks and implications associated with *N*. *dimidiatum* in various settings.

## 5. Understanding the Epidemiological Aspects of *Neoscytalidium dimidiatum*

### 5.1. Life Cycle of Neoscytalidium dimidiatum

The life cycle of *N. dimidiatum* presents intriguing dynamics, yet it remains a subject of ongoing research with several complex facets. While the epidemiology, including seasonal dynamics of different spore types of *Neoscytalidium*, is still inadequately understood, its impact on various crops and the occurrence of epidemics have been noted [11,27,77].

The interactions between this pathogen and its host plants are multifaceted and not fully elucidated [181]. Moreover, the virulence mechanisms employed by *N*. *dimidiatum* are still being explored.

As a plant pathogen, *N*. *dimidiatum* employs a dual strategy for asexual reproduction, as documented in various studies [2,50,51,52,53]. Firstly, it reproduces through hyphal fragmentation, where discrete fragments of the fungus detach, serving as vehicles for its dissemination to new host plants. Secondly, *N*. *dimidiatum* utilizes specialized structures known as fruiting bodies, within which it generates spores. These spores are subsequently released into the environment, poised for infecting new plant hosts. This intricate combination of asexual reproduction mechanisms equips *Neoscytalidium* with a remarkable capacity for efficient spread and establishment across diverse plant populations.

Crucially, *Neoscytalidium*’s adaptability underpins its propensity to initiate widespread diseases in agricultural and horticultural settings, presenting significant challenges in plant health management. It is worth noting that *N*. *dimidiatum* can infect its hosts through various means, including wounds and natural openings. Additionally, direct penetration can occur on specific host plants, such as dragon fruits, through the formation of appressoria, particularly on young tissues that are highly vulnerable to infection [45].

In conclusion, *N*. *dimidiatum*’s life cycle and pathogenicity represent intricate processes influenced by various factors, including host plants and environmental conditions. Further research is essential to fully comprehend the complexities of its interactions and the mechanisms underlying its adaptability as both a pathogen and a saprotroph.

### 5.2. Host Infection in a Unique Context of Dragon Fruit Canker

In the context of dragon fruit canker, acknowledged as the most destructive disease affecting dragon fruit plants, the fungal infection commences by generating appressoria on the surface, which is succeeded by a direct penetration into an epidermal cell. Initially, a swift collapse of chlorenchyma cells adjacent to the infection site occurs, accompanied by limited colonization of the tissue during the initial phases. In response to the infection, the host generates callus tissue, which forms around the impaired tissue, thereby curbing the progression of many infections. However, certain infections proceed to manifest extensive spreading lesions that contain embedded pycnidia. Internally, within the dead tissue, hyphal cells separate to give rise to arthrospores. Notably, lesion expansion is often preceded by the formation of prominent yellow halos, suggesting the production of a diffusible toxin by the pathogen. The existence of such a toxin is further corroborated by the chlorosis evident in cladode tissue subsequent to injection with cell-free culture filtrates of the fungus [45].

The most susceptible area for infection is the young tissue located directly behind the growing point of the cladode. The apexes of rapidly growing cladodes are particularly prone to infection. Throughout the inoculation phase, lesions are rarely observed beyond 3 cm from the apex. This specific susceptibility at certain sites explains the distinct bands of symptoms observed on mature cladodes in their natural environment. Each band represents infections that occurred during specific growth phases of the cladodes. Consequently, the highest vulnerability to infection in orchards corresponds to wet periods that coincide with the peak of vegetative growth. On the other hand, mature and aged cladodes demonstrate substantial resistance to infection [45].

Arthroconidia are confined within the necrotic tissues of the lesion and have limited dispersal capability, while pycniospores, released during wet weather and spread by rain, are the primary means of dispersal and infection. Therefore, the disease is more severe during the wet season when infection conditions are frequent [45]. While undocumented, there is a possibility that phragmospores formed on infected host surfaces could be disseminated by wind.

### 5.3. Factors Influencing Neoscytalidium dimidiatum Infection

#### 5.3.1. Factors Influencing Host Response

Pathogens belonging to the Botryosphaeriaceae family employ distinct modes of infection in plants, exploiting openings such as wounds in both green and woody tissues, as well as utilizing natural entry points in flowers, fruits, leaves, and green shoots. These pathogens employ enzymatic and toxic agents to induce cellular and tissue demise within the afflicted plant organs. In the context of perennial woody hosts, incursions occurring within the inner layers of the wood or immediately beneath the bark can inhibit shoot development, culminating over time in the eventual withering of shoots, commonly referred to as ‘dieback’ [182].

*Neoscytalidium dimidiatum*, much like other species within the Botryosphaeriaceae family, is categorized as a stress-associated pathogen. Disease manifestation is frequently intertwined with the presence of abiotic stressors, including factors such as drought, physical damage, and unfavorable growing conditions, as substantiated by various studies [33,40,41,46,76,183,184]. The diseases induced by *N*. *dimidiatum* predominantly coincide with the onset of these stress factors, which are independent of the *N*. *dimidiatum* infection itself. In such scenarios, *N*. *dimidiatum* actively contributes to the emergence of observed symptoms, albeit to varying extents, contingent upon the severity and duration of the stress conditions. Notably, these disease symptoms can escalate swiftly, leading to extensive losses over expansive geographic regions when the causative stress factor is pervasive. Furthermore, the convergence of multiple factors, including biological pressures from pathogens and pests that are extending their geographic ranges, may collectively contribute to the conducive environment for the development of *N*. *dimidiatum*-induced diseases, as postulated by Slippers and Wingfield [185].

Across a diverse range of woody hosts in tropical and semi-tropical regions, *N*. *dimidiatum* functions as a wound-invading pathogen, with a tendency to primarily infect branches and trunks that have experienced damage from sunburn, freezing, or pruning [170]. Its primary focus of infection lies in the cambial region of the tree, leading to symptoms of wilting and branch dieback [186,187]. Subsequently, blisters and fissures develop on the bark, revealing black spore masses that are dispersed by wind. Concurrently, the affected wood exhibits a distinctive brown staining. When introduced into bark wounds extending to the vascular cambium or exposed xylem due to pruning, the pathogen demonstrates the capacity to induce canker formation [165]. These cankers primarily display an annual rather than perennial nature. The pathogen is primarily localized within the cell lumens of the xylem and phloem, with intercellular spread primarily occurring through pits. However, the excision of cankers, whether or not a wound protectant is applied, has proven ineffective in managing the disease [165]. However, pruning and grafting wounds are identified as the probable primary entry point for infection [76,138]. The application of appropriate fungicides or biological control agents to protect these wounds still may have a chance to prevent or minimize the occurrence of such infections. In the study conducted by Çiftçi et al. [138], it was observed that the regrafting of unproductive walnut trees with Chandler, when they were older than 8 to 10 years, resulted in the frequent and severe development of canker symptoms. This practice leads to tree loss in the affected orchards.

In their recent study, Güney et al. [76] observed that cracked tree trunks, branches, and limbs represent the most notable symptoms of canker in all examined olive cultivars. However, the severity and variety of symptoms were significantly influenced by multiple factors, including the cultivar, age of the tree, season, and biotic and abiotic stress factors. While trees of all ages are affected, younger trees were particularly impacted, resulting in higher mortality rates. Symptoms were most noticeable during the summer and fall seasons. Pruned trees, those with injury, ripe fruits, and mechanically injured plant parts appeared to be more susceptible to the pathogen, exhibiting large canker formations or black sporulation on exposed areas. Dying leaves, twigs, branch cankers, and fallen leaves and fruits all play a role in the disease cycle and the survival of the pathogen. Water deficiency and drought were recognized as significant stress factors that increase susceptibility, leading to the exacerbation of disease severity. Yeganeh and Mohammadi [39] investigated the production of loose spores by *N*. *dimidiatum* beneath the bark of infected banyan trees and in the pruning debris. This observation aligns with prior research that Botryosphaeriaceae species, known for inducing trunk diseases in fruit and forest trees, can also exist as saprophytes within wood debris [1]. These species can persist and produce spores in wood debris, serving as crucial sources of inoculum for infections in woody plants.

Gusella et al. [41] investigated various aspects of *N*. *dimidiatum*’s behavior. Their findings revealed that older branches (>3 years old) exhibit a longer timeline for canker development compared to their younger counterparts. They also made an intriguing observation about the absence of *N*. *dimidiatum* within fruit mummies, which are inhabited by various other fungal saprophytes.

#### 5.3.2. Climatic and Edaphic Factors

Holland et al. [32] document that the timing of inoculation significantly impacts the development of *N*. *dimidiatum*-induced lesions on almond trees. Lesions are more pronounced when inoculation occurs during the summer compared to smaller lesions resulting from winter inoculation. These findings underscore the pathogen’s adaptability to elevated temperatures, as exposure to high temperatures can lead to substantial plant damage. Researchers such as Gusella et al. [75], Güney et al. [76], Hohenfeld et al. [73], Hong et al. [11], Nouri et al. [30], and Sabernasab et al. [74] have consistently reported the optimal temperature range for *N*. *dimidiatum*’s growth, sporulation, conidial germination, and pycnidia formation to be between 30 and 36 °C. Notably, Güney et al. [49] have conducted in vitro analyses, revealing that conidial germination reaches its zenith at 40 °C. Likewise, Coutinho et al. [72] demonstrated that *Neoscytalidium* is capable of thriving at a high temperature of 40 °C. Hohenfeld et al. [73] have particularly concentrated on the black root rot induced by *N*. *dimidiatum* on cassava, elucidating that the temperature range of 32–36 °C yields the highest disease severity. Hassan et al. [188] observed that the susceptibility of saplings to stem canker caused by *N*. *dimidiatum* is influenced by the duration of warm temperatures and the abundance of fungal inoculum. To further explore the impact of temperature conditions on canker development, a growth chamber was utilized with two temperature regimes: a very hot condition at 40 °C and a hot condition at 32 °C. Among the pre-inoculation regimes, the very hot and hot temperatures were identified as the most favorable for infection in eucalyptus saplings when compared to other host species. Mayorquin et al. [20] conducted a temperature study on lesion development caused by the pathogen, supporting these findings by showing that increased temperatures lead to more aggressive infection by this pathogen. Machado et al. [118] and Adesemoye et al. [171] observed that *N*. *dimidiatum* causes disease in semi-arid and desert regions, while its presence is absent in more humid areas. These findings and observations further accentuate the detrimental effects of elevated temperatures on plant vitality, indicating the pathogen’s thermotolerant nature.

Gusella et al. [41] demonstrated in laboratory experiments that *N*. *dimidiatum* efficiently colonizes desiccated fig fruits within a temperature range of 20 °C to 35 °C. Additionally, their in vitro studies on water potentials reveal a direct correlation between the mycelial growth rate and decreasing water potential (ranging from 1 to 3 MPa), with the specific type of salt used further influencing this reduction. In a separate study by Kuan et al. [184], they extensively explored the metabolic attributes of *N*. *dimidiatum*, including nutritional profiling, pH tolerance, and osmotolerance. Their research emphasized that the fungus exhibits optimal growth within a pH range of 4.5–5.5, highlighting the importance of future investigations into substrate utilization under varying growth conditions.

### 5.4. Mode of Transmission

Similar to other members of Botryosphaeriaceae, the dissemination of this pathogen occurs through various means such as wind, rain, and diverse arthropod groups [39,165,176,186,187,189]. Jiménez Luna et al. [48] carried out a comparison of fungal community compositions obtained from spore traps and those acquired from plant samples collected from symptomatic almond and walnut trees. Their study consistently confirmed the identification of four distinct species, including *N*. *dimidiatum*, using both methodologies across various orchards. This discovery highlights the crucial role of airborne spores in the epidemiology of *N*. *dimidiatum* and strengthens the reliability and efficacy of these detection techniques. During our pathogenicity assays involving *N*. *dimidiatum* on various plant species, we made an unpublished observation of viable plant colonization by dust particles originating from air conditioner filters (personal communication with our research team member Şahimerdan Türkölmez). This occurrence is attributed to direct and prolonged exposure to air-conditioning and air-circulating systems within our growth rooms. Interestingly, Heidari et al. [3] conducted a study focusing on *N*. *dimidiatum* within human respiratory tracts. The ability of *N*. *dimidiatum* to thrive in such diverse environments, ranging from plants to human tissues, emphasizes its adaptability and raises questions about its potential impact on both human and plant health.

Al-Sadi et al. [46] emphasized the significance of nurseries and the potential spread of the pathogen through planting infected nursery plants. Türkölmez et al. [47] suggested that *N*. *dimidiatum* can potentially be transmitted through seeds, soil, and/or the air, and it may persist in the soil in association with infected tomato debris. While Mirtalebi et al. [107] and Güney et al. [49] have demonstrated the pathogen’s capacity to colonize the seed coat, the potential for *N*. *dimidiatum* to spread through the seeds of its host plants remains uncertain. Yeganeh and Mohammadi [39] have provided evidence that the pathogen’s conidia can also be transported by arthropods. In their study, Zhu et al. [189] successfully isolated *N*. *dimidiatum* from the beetle gallery system within infested elm (*Ulmus densa*) stems in China. This finding suggests the potential occurrence of *N*. *dimidiatum* within beetle galleries, a common habitat for various fungi, highlighting the ecological relevance of *N*. *dimidiatum* in this specific context. Considering that the beetle assists in carrying its conidia, it may indirectly aid in their dissemination. Avian species, rodents, and other small animals may serve as potential vectors for disseminating the pathogen through infected fruits and seeds [190].

During a survey on mango conducted by Ray et al. [35], *N*. *hyalinum* was also isolated from nearby fig shrubs (*Ficus carica*), indicating the potential movement of the pathogen between various plant hosts. Esmaeili et al. [98] observed canker and severe dieback symptoms on barberry trees in specific areas of Fars Province, Iran. Some of these trees were situated in vineyards that had been previously studied for grapevine trunk diseases. The researchers aimed to investigate whether barberry trees could serve as hosts for fungal trunk pathogens affecting grapevines. They collected wood samples from infected barberry trees and isolated fifteen fungal species, including *Phaeoacremonium* spp., *D*. *seriata*, *N*. *dimitatum*, and *B*. *dothidea*. The pathogenicity tests on both barberry and grapevine revealed that the isolates collected from barberry trees demonstrated cross-pathogenicity on grapevines. Their study underscores that barberry trees can act as alternative hosts for diverse grapevine fungal trunk pathogens, including *N*. *dimidiatum*.

## 6. Insights into the Virulence and Genomic Characteristics of *Neoscytalidium dimidiatum*

### 6.1. Host–Pathogen Interaction at Molecular Level

The virulence of Botryosphaeriaceae pathogens relies on an array of molecules known as effectors, including enzymes responsible for cell wall degradation, secondary metabolites, and peptidases [191]. Next-generation sequencing (NGS) has revolutionized fungal research within the Botryosphaeriaceae family, revealing unique gene and repeat distributions linked to co-expression patterns [192]. These pathogens employ a multifaceted biochemical arsenal to manipulate host physiology. This includes the synthesis of polyketide toxins like terremutin and mellein, known for their roles in defense mechanisms and necrosis induction [193,194]. They also rely on extracellular proteins [195], secreted polysaccharides [196], and jasmonic esters [197] to facilitate pathogenicity. Central to their arsenal are carbohydrate-active enzymes (CAZymes), particularly glycoside hydrolases (GH), which enable the degradation of wood components like cellulose and hemicelluloses [182,192,198]. While GH enzymes hold biotechnological promise and are potential phytoprotection targets, their precise in vivo mechanisms remain only partially understood. Other CAZyme categories include glycosyltransferases, carbohydrate esterases, polysaccharide lyases, and auxiliary activities, often associated with carbohydrate-binding modules [199]. The current understanding of proteases produced by Botryosphaeriaceae and their likely vital roles in fungal physiology and pathogenesis is very limited [192,198].

In recent years, *N*. *dimidiatum* has garnered increased attention through extensive research endeavors. Despite its importance, the population genetics of *N*. *dimidiatum* remains relatively unexplored, with limited studies providing insights into its virulence and genomic characteristics [182,184,191]. This pathogen exhibits significant variation in virulence across species within the Botryosphaeriaceae family, with unique attributes, such as mannanases, α-glucosidase enzymes, and pectate lyases, setting *N*. *dimidiatum* apart. These unique enzymatic traits, especially GH76 and PL3 proteins, are notably prevalent in *N*. *dimidiatum* compared to other species [182].

Genomic analyses have unveiled putative coding DNA sequences, including genes related to melanin biosynthesis, a known contributor to fungal virulence and adaptation to diverse environments. These analyses also reveal genes associated with iron uptake, which is crucial for various fungal cellular processes. The fungus displays a set of CAZymes linked to plant pathogenicity, involving the decomposition of hemicellulose and pectin [184].

Moreover, investigations into host–pathogen interactions have identified key genes associated with plant defense responses and calcium ion signaling pathways in red-fleshed pitaya [181,200]. These studies emphasize the potential roles of specific genes, including PR homologous proteins and WRKY transcription factors, in regulating pitaya’s defense mechanisms against *N*. *dimidiatum* infection. Transcriptomic analyses have also revealed the presence of Leucine-Rich Repeat (LRR) genes, primarily within the LRR-RLK, NBS-LRR, and FBXL subfamilies, underscoring their involvement in plant defense mechanisms against *N*. *dimidiatum*. These genetic insights contribute to our understanding of the molecular responses of pitaya to *N*. *dimidiatum* infection, highlighting potential key players in plant defense. Notably, research by Wang et al. [201] on reference genes for accurate gene expression analysis in *N*. *dimidiatum* offers valuable guidance for future studies, facilitating more precise investigations into the gene expression dynamics of this pathogen under varying conditions.

Collectively, these studies enhance our understanding of *N*. *dimidiatum*’s biology, genetics, and interactions with host plants. Continued research in this area holds the promise of further elucidating the mechanisms underpinning its pathogenicity and host interactions, ultimately aiding in the development of effective strategies for disease management and control.

### 6.2. Neoscytalidium dimidiatum Virulence in Diverse Host Plants

In the intricate realm of plant–pathogen interactions, understanding the virulence and impact of fungal species becomes paramount for effective disease management. *N*. *dimidiatum*, a member of the Botryosphaeriaceae family and other canker-causing fungi, has garnered considerable attention from diverse research teams for its aggressive tendencies and far-reaching consequences on various hosts. Through a series of studies, researchers have delved into the complexities of this fungal species, shedding light on its behavior, impact, and virulence. The virulence of *N*. *dimidiatum* stands out as a central theme across these investigations, positioning it as one of the most aggressive species within its family. The research by Marques et al. [84] into Botryosphaeriaceae fungi associated with mango dieback and stem-end rot in Northeastern Brazil yielded valuable insights. Their exploration identified seven taxa, including *N*. *dimidiatum*. Inoculation experiments on mango fruits highlighted the potential of various Botryosphaeriaceae species to induce damage, with *N*. *dimidiatum* and *Neofusicoccum parvum* exhibiting the largest lesions, underscoring their elevated virulence. Mayorquin et al. [20] conducted a study to identify the fungi associated with citrus branch canker and dieback disease in the desert areas of southern California. *N*. *hyalinum*, *Eutypella citricola*, *E*. *microtheca*, and an unidentified species of *Eutypella* were identified. *N*. *hyalinum*, the most often recovered fungus, together with *Eutypella* sp., induced much longer infections than other *Eutypella* species on ‘Lisbon’ lemon branches. Panahandeh et al. [156] contributed insights through their investigation into pathogenicity tests on different fungal species inoculated into *Syzygium cumini* shoots. Among the nine species examined, *N*. *hyalinum* emerged as a prominent contributor, inducing notably elongated lesions, thus unveiling its distinct impact on the host. A study by Kazemzadeh et al. [1] centered on pathogenicity assessments of nine Botryosphaeriaceae species in diverse forest trees in Iran. Their evaluation highlighted significant disparities in virulence among species, with *N*. *novaehollandiae*, *Botryosphaeria dothidea*, and *Diplodia intermedia* emerging as particularly aggressive agents. Notably, *N*. *novaehollandiae* exhibited heightened virulence on multiple host species, showcasing its adaptability to diverse environments. Ghasemi-Sardareh et al. [145] focused on the identification of fungal species associated with trunk diseases in neem trees (*Azadirachta indica*). Among an array of identified fungal taxa, *N*. *hyalinum* emerged as a potent contributor, displaying a high level of aggressiveness substantiated by the extent of vascular necrosis observed within the wood. In a study conducted by Çiftçi et al. [138] using walnut seedlings cv. Chandler, *N*. *dimidiatum* exhibited greater virulence than *Lasiodiplodia theobromae*, resulting in longer necrotic lesions.

However, contrasting outcomes have arisen concerning the most virulent species across distinct hosts. Chen et al. [136] conducted a pivotal study, isolating ten Botryosphaeriaceae family species, including *N*. *dimidiatum*, from varied walnut infections in California. Pathogenicity assessments involving English walnut cultivars (‘Chandler’, ‘Tulare’, and ‘Vim’) demonstrated the pathogenicity of all species, with *L*. *citricola* and *N*. *parvum* exhibiting the highest pathogenicity, followed by *N*. *mediterraneum*, *N*. *dimidiatum*, and *B*. *dothidea*. Exploring Bot gummosis in California citrus, Adesemoye et al. [171] investigated Bot gummosis infections in California’s citrus crops. In experiments using Eureka lemon shoots, lesion lengths varied based on the employed isolates. Particularly, *Neofusicoccum luteum* isolates induced longer lesions, while *Dothiorella viticola* isolates resulted in shorter lesions compared to other species, including *N*. *dimidiatum*. Hashemi and Mohammadi [43] studied the decline in willow and poplar trees in Iran, identifying nine fungal species linked to the decline symptoms, including *N*. *hyalinum*. Pathogenicity tests demonstrated that *L*. *theobromae* caused the longest lesions on willow, while *Phaeoacremonium parasiticum* induced the longest lesions on poplar. Further, Hashemi et al. [176] addressed elm tree decline, highlighting *Dothiorella sarmentorum* as the most virulent species based on wood necrosis extent. Coutinho et al. [72] examined Botryosphaeriaceae species associated with cashew, mango, and guava dieback, stem, and branch cankers. All isolates, including *N*. *hyalinum*, showed virulence, with *Neofusicoccum* strains being the most aggressive in mango, cashew, and ‘caja-umbu’ (*Spondias mombin* × *S*. *tuberosa*) plants. Espargham et al. [168] found that *L*. *theobromae* was the most virulent species on lime shoots, while Yeganeh and Mohammadi [39] reported the same for banyan trees compared to *N*. *dimidiatum* and other species. Brito et al. [122], in a Brazilian study on cassava investigating root and stem dry rot pathogens, *N*. *dimidiatum* and nine *Lasiodiplodia* species were analyzed. *Lasiodiplodia laeliocattleyae* exhibited the highest virulence, followed by *N*. *dimidiatum* and *L*. *parva*. Other species showed lower virulence levels, with no significant differences among them. Arkam et al. [202] investigated the Botryosphaeriaceae family, a fungal group with a global impact on grapevine production, under Algerian conditions. They identified eleven species from six genera, including *N*. *dimidiatum*. Pathogenicity trials involving grapevine green shoots showed that all identified species exhibited pathogenicity, with *Neofusicoccum parvum* and *L*. *theobromae* being the most aggressive. On barberry, *L*. *theobromae* exhibited a higher level of aggressiveness than *N*. *dimidiatum* on both barberry and grapevine [98]. In pomegranate, lesions induced by *L*. *theobromae* were significantly longer, whereas *N*. *dimidiatum* caused larger and deeper wood lesions compared to other species [144].

Ahmadpour et al. [40] reported notable variations in the response of different host species upon the reintroduction of *N*. *novaehollandiae* to its original substrates. It is worth mentioning that *Conocarpus erectus*, *Eucalyptus camaldulensis*, and *Morus nigra* had an increased predisposition towards severe wood discoloration accompanied by the appearance of a characteristic black powdery manifestation. In contrast, both *Ficus religiosa* and *Bauhinia purpurea* exhibited a somewhat reduced degree of these phenotypic modifications. These findings align harmoniously with the outcomes of concurrent field evaluations, thereby corroborating the presence of divergent patterns in disease incidence and severity within the ambit of the examined arboreal taxa.

In conclusion, the multifaceted investigations into *N*. *dimidiatum* and the Botryosphaeriaceae family have unveiled the complex nature of pathogenicity and virulence. The dynamic interactions between these fungi and their hosts, coupled with the intricate genetic makeup, provide a deeper understanding of their behavior and impact. These insights are crucial for devising effective disease management strategies and safeguarding the health of diverse plant species. As the body of research grows, the intricate web of interactions between fungi, hosts, and the environment continues to unravel, offering valuable perspectives on combating plant diseases and ensuring sustainable agricultural practices. The ongoing development of NGS platforms and their integration with other emergent technologies hold the promise of enhancing our understanding of its biology and evolution [192].

## 7. Management Strategies for *Neoscytalidium dimidiatum*

### 7.1. Antifungal Activity of Different Applications

*Neoscytalidium dimidiatum* poses risks to various crops, notably dragon fruit, causing stem canker and fruit rot. Traditional chemical solutions, though effective, have drawbacks. This prompts researchers to explore alternative approaches to combat this fungal threat and reduce its impact on agriculture. This review section delves into recent studies examining *N*. *dimidiatum*’s susceptibility to interventions such as fungistatic pesticides, essential oils, biopesticides, biocontrol agents, and nanoparticles. This exploration aims to uncover optimal methods for tackling this fungal challenge and minimizing its effects on crop health.

#### 7.1.1. Fungicides

Xu et al. [12] conducted groundbreaking research that highlighted the susceptibility of *N*. *dimidiatum* to a range of fungistatic pesticides, notably including hexaconazole, tebuconazole, flusiazole, and pyraclostrobin. Of particular significance is the potency demonstrated by pyraclostrobin EC at concentrations of 250 g/L, resulting in commendable control rates reaching up to 85%, comparable to the efficacy of azoxystrobin SC and tebuconazole. This insight was further corroborated by Xian et al. [203], who confirmed that field applications of pyraclostrobin EC at similar concentrations effectively reduced disease incidence to 85%, emphasizing the practical utility of this approach for stem canker control.

Concurrently, Noegrahati et al. [204] introduced azoxystrobin (200 g/L) and difenoconazole (124 g/L) as potent agents for managing stem canker in red dragon fruit. Their research underscores the safety of these agents in terms of residue, ensuring their viability for consumption. Parallelly, Eraslan Sür and Oksal [205] delved into the effects of fungicide concentrations on *N*. *dimidiatum* isolated from apricots. The synergistic combination of Floupyram (200 g/L) with Tebuconazole (200 g/L), along with Cyprodinil + Fludioxonil (37.5 + 25%) fungicides, demonstrated robust suppression of mycelial growth, accentuating their pivotal role in mitigating the expansion of this pathogenic fungus.

Fullerton et al. [45], in collaboration with the Southern Horticultural Research Institute (SOFRI), identified efficacious fungicides for canker control. This includes protectant fungicides like mancozeb and copper, as well as active constituents such as difenoconazole and azoxystrobin. These agents, with their translaminar and curative attributes, effectively permeate cladodes and counter the fungus post-infection. Fullerton et al. [45] underscored the importance of adaptive strategies in response to climate, recommending curative fungicides during wet seasons marked by frequent infections and protective fungicides timed with forecasted rainfall in dry seasons.

In parallel, the rigorous study conducted by Al Raish et al. [127] unveiled the vulnerability of royal poinciana trees in the UAE to stem canker disease caused by *N*. *dimidiatum*. This affliction encompasses symptoms such as desiccated branches, wilting leaves, bark lesions, xylem discoloration, wood necrosis, and gumming. Through rigorous investigation, diverse chemical fungicides, including Protifert^®^, Cidely^®^ Top, and Amistrar^®^ Top, were assessed. Significantly, Cidely^®^ Top emerged as the optimal treatment against N. dimidiatum in field conditions, demonstrating its potential significance in managing the impact of this pathogen on royal poinciana trees.

Riska et al. [206] conducted in vitro experiments revealing that sodium salt, particularly when concentrations exceeded 3%, displayed substantial potential in hindering the mycelial growth of *Neoscytalidium* isolates. Furthermore, through a field trial, the application of a sodium salt solution at 30 g/L twice a week, combined with the rotation of chemical fungicides (propineb, 80% mancozeb, 50% carbendazim, and difenoconazole 250 g/L) once a week, effectively reduced the severity of stem canker disease on dragon fruit plants. The collective findings from culture and field investigations provide support for the feasibility of utilizing alternating chemical fungicides as a prospective strategy for managing stem canker disease on dragon fruit.

#### 7.1.2. Essential Oils and Biopesticides

The efficacy of essential oils in addressing *N*. *dimidiatum* remains relatively uncharted. Campos-Rivero et al. [207] introduced a novel strategy involving active sachets encapsulating oregano oil with starch/agave fructans. These sachets, endowed with antifungal attributes, aim to combat postharvest losses caused by phytopathogens in fruits and vegetables. Through in vitro experiments, their study demonstrated the efficacy of oregano oil sachets in significantly suppressing the growth of *N*. *hyalinum* (now *N*. *dimidiatum*) at a temperature of 30 °C over a span of 12 days. This investigation marks the inaugural exploration of the potential of gaseous oregano oil to impede mycelial expansion in *N*. *dimidiatum*.

In addition, the study conducted by Taguiam et al. [208] demonstrates the inhibitory capacity of citronella oil, exhibiting a range of effects from 60% to 85.42% against *N*. *dimidiatum*. The same authors highlight a biopesticide formulation containing *Bacillus subtilis*, mancozeb, isoprothiolane, and citronella essential oil as a promising solution for mitigating *N*. *dimidiatum*. Nevertheless, given the partial inhibition of fungal growth by citronella oil (1.25 µL/mL), a thorough assessment of its potential phytotoxicity on dragon fruits is imperative before considering field applications. In Ratanaprom et al.’s [209] study, their experiments demonstrated that the combination of *B*. *subtilis* supernatant and sodium bicarbonate effectively inhibited the growth of *N*. *dimidiatum* through both direct and indirect mechanisms. The direct effect involves the secretion of antibiotic metabolites into the growing medium. Simultaneously, this treatment triggers the defense mechanisms of dragon fruit plants, leading to increased levels of PR proteins and enzymes associated with phenylpropanoid biosynthesis. This dual-action approach offers a robust defense against stem brown spot disease in dragon fruit plants.

Furthermore, Balendres et al. [13] demonstrated that the in vitro growth of *N*. *dimidiatum* can be effectively inhibited by a bio-fungicide formulation comprising *B*. *subtilis*, isoprothiolane, and mancozeb. This finding underscores the potential of the bio-fungicide in managing the growth and spread of *N*. *dimidiatum*. Such efficacy could prove invaluable for devising disease-management strategies in agricultural contexts, with potential implications for postharvest fruit handling practices and future investigations into the management of dragon fruit diseases.

#### 7.1.3. Biocontrol Agents

Wang et al. [210] investigated the potential of *Penicillium rolfsii* (strain Y17), an endophytic fungus isolated from papaya leaves, as a control agent against *N*. *dimidiatum*-induced pitaya fruit canker disease. They systematically assessed the impact of Y17 treatment on disease progression, internal defense enzymes, and malondialdehyde content within the fruit. The results unveiled that Y17 treatment prompts the activation of antioxidant enzymes, thereby ameliorating oxidative stress and mitigating disease-associated damage. Added to that, this treatment fortifies the fruit’s immune responses and resistance to infection, offering promising avenues for advancing novel strategies in countering pitaya fruit canker disease.

Al Hamad et al. [211] examined the potential of actinobacterial isolates as biological control agents (BCAs) for managing stem canker disease on royal poinciana, attributed to *N*. *dimidiatum*. Their investigation highlights the inhibitory prowess of *Streptomyces griseorubens* UAE2 and *Streptomyces wuyuanensis* UAE1. These strains showcase robust antifungal effects through the production of antifungal compounds and cell-wall-degrading enzymes. Notably, *S*. *griseorubens* exhibits an added dimension by reinstating the activity of 1-aminocyclopropane-1-carboxylate deaminase (ACCD). Greenhouse and container nursery trials demonstrate the efficacy of *S*. *griseorubens* in curtailing disease symptoms, evidenced by reduced conidia numbers and defoliated leaves among royal poinciana seedlings. Their study underscores the superiority of *S*. *griseorubens* as a BCA, attributed not only to its antifungal attributes but also its ACCD secretion, contributing to effective stem canker disease management.

Lin et al. [212] focused on the efficacy of biocontrol strains for combatting pitaya canker. Their investigation centered on bacterial strain AF01, identified as *Paenibacillus polymyxa*. AF01 displays potent antifungal effects against *N*. *dimidiatum* and other pitaya fungal pathogens, attributed to the production of 13 fusaricidins that impair mycelial growth and spore germination by disrupting fungal cell membranes and ultrastructure. Pot experiments and yield evaluations validate AF01′s capacity to curtail the disease index. RNA-seq analysis provides insights into AF01′s targeted disruption of cellular processes, including cell wall biosynthesis and protein biosynthesis. These revelations suggest that *P*. *polymyxa* AF01 holds promise as a biocontrol agent for pitaya canker.

Ma et al. [213] identified bacterial strain P42 as *Lysinibacillus boronitolerans*, demonstrating robust antagonistic effects (82.18%) against soilborne pathogens. Strain P42 showcased broad-spectrum antagonistic activity encompassing *N*. *dimidiatum* and other tropical agricultural fungal pathogens.

In Travadon et al.’s [214] study, they assessed *Trichoderma*-based Biological Control Agents (BCAs) in a laboratory using detached almond stems. These evaluations aimed to determine the BCAs’ effectiveness against four canker pathogens, including *N*. *dimidiatum*. Their findings highlighted the remarkable success of two specific *Trichoderma* strains, *Trichoderma atroviride* SC1 and *T*. *paratroviride* RTFT014, in significantly reducing infections caused by all canker pathogens in almond trees.

#### 7.1.4. Nanoparticles

Cu_2_O-Cu nanoparticles stabilized by alginate, developed by Du et al. [215], serve as a potential plant fungicide against *N*. *dimidiatum*-induced brown spot disease in dragon fruit plants. Cu_2_O-Cu nanoparticles stabilized in alginate exhibit noteworthy antifungal efficacy (~100%) against *N*. *dimidiatum* at a copper concentration of 30 ppm. This points to the substantial potential of Cu_2_O-Cu nanoparticles in alginate as promising nanomaterials for plant fungicide applications.

Tuan et al. [216] investigated the management of brown spot disease in dragon fruit plants caused by *N*. *dimidiatum*. They explored the efficacy of plant elicitors, oligochitosan (OC) and nanosilica (nSiO_2_), and prepared OC and nSiO_2_ synthesized from rice husk. The combined nSiO_2_ and OC treatment, referred to as nSiO_2_-OC, demonstrated efficacy in enhancing chitinase induction and reducing disease severity. Their study highlights the potential of utilizing the nSiO_2_-OC hybrid material as an innovative elicitor for brown spot disease management in dragon fruit plants, offering environmentally sustainable alternatives to hazardous agrochemicals.

Acay et al. [217] introduced a cost-effective method to synthesize a chitosan bionanocomposite using *Morchella esculenta* extract. The synthesized *M*. *esculenta*-chitosan bionanocomposite exhibited antifungal properties against various plant pathogens, including *N*. *dimidiatum*.

Hashem et al. [218] assessed the efficacy of a clove essential oil nanoemulsion (CEONE) as a control agent against *N*. *dimidiatum* affecting *Carum carvi* plants. CEONE exhibited substantial antifungal activity (25.5% to 82.2% inhibition) against *N*. *dimidiatum*. In pot experiments, CEONE and clove essential oil (CEO) mitigated *N*. *dimidiatum* blight disease, enhancing root length, plant height, and leaf development. Their study also explored CEONE and CEO’s potential to induce plant resistance through changes in biochemical markers.

Duong et al. [219] evaluated the antifungal efficacy of green-synthesized Cu/Cu_2_O nanocomposites against *N*. *dimidiatum* using in vitro assays. These nanocomposites exhibited significant antifungal potency against *N*. *dimidiatum*, with a minimum inhibitory concentration (MIC) of 0.0625 g/L and a measured inhibition zone diameter of 18.00 ± 0.58 mm. These findings highlight their potential utility as effective antifungal agents against *N*. *dimidiatum*.

### 7.2. Strategies for Control

In order to ensure the successful control of *N*. *dimidiatum*, it is imperative to give careful attention to the numerous biological and technical factors that contribute to its multidimensional nature. Central to effective disease mitigation is the comprehension and resolution of latent infections, where *N*. *dimidiatum* invades host plants and products inconspicuously. Sole reliance on visual inspection for latent infection detection proves inadequate, complicating pathogen identification and management [45,122,220]. In addition, the visual similarity between symptoms attributed to *N*. *dimidiatum* and those caused by other species within the *Fusicoccum* or *Scytalidium* genera, along with Botryosphaeriaceae fungi, hinders precise differentiation and risks misdiagnosis [45,122]. The absence of rapid molecular diagnostic tools further compounds the challenges, with in planta identification methods currently lacking practicality [45].

The vast host range of *N*. *dimidiatum* magnifies the complexity of control efforts, necessitating tailored identification and management strategies due to the heterogeneous susceptibility across species [122]. Amidst these challenges, investing in comprehensive research and development emerges as paramount. The establishment of swift and reliable diagnostic methodologies rooted in molecular techniques stands to greatly enhance early pathogen detection [45]. Advancing our understanding of *N*. *dimidiatum*’s biology, host interactions, and disease progression holds promise for formulating precise control strategies tailored to specific host plants [45,122].

Fullerton et al. [45] emphasize the significance of rigorous orchard hygiene as a fundamental principle for the successful management of canker disease. Vital to successful control is the elimination of spore sources and the prompt removal of infected cladodes, while the time lag between symptom manifestation and sporulation offers an opportunity for pre-emptive excision of nascent lesions. Combining robust orchard hygiene with judicious fungicide application proves instrumental in expeditiously reducing disease burden, facilitating orchard recovery within a matter of months.

Brito et al. [122] accentuated the centrality of stem-cutting dry rot in the survival and dissemination of pathogenic Botryosphaeriaceae within cassava. Effective management strategies for black root rot and stem-cutting dry rot necessitate the utilization of high-quality propagating material and precise pretreatment protocols. Sakalidis et al. [83] identified *N*. *novaehollandiae* and *N*. *dimidiatum* among various Botryosphaeriaceae taxa with diverse roles as canker-associated fungi, endophytes, or potential pathogens. The unique growth conditions of the Kimberley region hinder pathogen establishment, while mechanical pruning wounds and excessive pruning contribute to plant stress.

In light of *N*. *dimidiatum*’s primary entry through wounds, especially those incurred during mechanical pruning, the mitigation of such wounds through careful practices or, where possible, avoiding pruning altogether becomes pivotal [76,165]. The application of fungicides or the introduction of effective biological control agents targeted at wound sites, such as pruning and grafting wounds, emerges as a promising preventive measure [76,138]. Targeted fungicide application during wet periods when infection conditions are conducive may further bolster control efforts [45]. Exploration of host cultivars exhibiting resistance or reduced susceptibility to *N*. *dimidiatum* canker provides valuable insight for breeding programs aimed at developing resistant varieties [122]. Ray et al. [35] and Esmaeili et al. [98] emphasized the significance of incorporating susceptible host functions into strategies for managing trunk diseases.

Owing to the need to minimize inoculum sources, orchard hygiene continues to be a pillar, necessitating the careful elimination and appropriate disposal of infected plant debris [45]. Given *N*. *dimidiatum*’s affinity for warmer temperatures, careful management of orchard temperature conditions, including irrigation and shading practices, warrants consideration [32]. By implementing rigorous protocols, conducting thorough inspections, and collaborating with experts, nurseries can significantly reduce the risk of propagating this pathogen and other latent infections in agricultural and horticultural settings [46]. The careful selection of seeds from reliable sources is also crucial in preventing the spread of *N*. *dimidiatum* [47,49,107].

A holistic approach to control involves vigilant monitoring for early signs of infection and employing rapid molecular-based diagnostic methods [45,48]. Research into the potential vectors, such as seeds and arthropods, aids in understanding disease dissemination [39,49,107,190]. Ongoing research into *N*. *dimidiatum*’s behavior, interactions, and disease progression contribute critical insights to developing refined control strategies [33,41,45].

With these research insights, investing in comprehensive research and development assumes pivotal importance for effective *N*. *dimidiatum* control. Molecular-based diagnostics and an enhanced understanding of pathogen–host dynamics offer the potential for targeted control strategies aligned with specific host plants. Integrating these strategies into disease management protocols promises to bolster crop yields and secure the sustainability of agricultural practices.

## 8. Future Challenges

In light of the comprehensive investigations into *N*. *dimidiatum*’s taxonomy, host range, biology, virulence traits, and management strategies, several intriguing avenues for future research emerge. While some progress has been made in understanding *N*. *dimidiatum*’s genetic makeup, there remains a need to unravel the species’ specific genetic traits linked to its adaptation to diverse environments. Uncovering the genetic mechanisms that allow *N*. *dimidiatum* to thrive across various hosts and ecological niches could shed light on its remarkable adaptability. Elucidating the intricate dynamics between *N*. *dimidiatum* and its host plants holds considerable potential. Exploring how the pathogen interacts with plants at the molecular, cellular, and physiological levels could unveil the underlying mechanisms driving infection, colonization, and disease development. Given the potential impact of climate change on *N*. *dimidiatum*, future research should delve into how shifting climatic conditions might influence the distribution, prevalence, and severity of *N*. *dimidiatum* infections. Understanding these dynamics can aid in developing targeted management strategies. As the management of *N*. *dimidiatum* and related pathogens remains a challenge, exploring novel, ecofriendly approaches could prove fruitful. Investigating the potential of biological control agents, as well as eco-friendly chemicals, could offer sustainable alternatives for disease management. Understanding how human-related factors, such as urbanization and agricultural practices, may influence *N*. *dimidiatum*’s ecology could provide insights into effective management strategies. Investigating any potential shifts in the pathogenicity or virulence of *N*. *dimidiatum* strains is essential, especially in light of changing environmental conditions and potential interactions with other pathogens. This research can enhance our ability to predict and mitigate disease outbreaks.

In conclusion, the intricate web of interactions involving *N. dimidiatum* necessitates continued research to unravel its genetic, ecological, and pathogenic intricacies. Addressing these future challenges will not only enhance our understanding of this enigmatic pathogen but also contribute to the development of effective strategies for disease management and mitigation.

## Data Availability

All relevant data generated or analyzed during this study are included in this manuscript.

## Supplementary Materials

The following supporting information can be downloaded at: https://www.mdpi.com/article/10.3390/jof9111048/s1, Table S1: Collection details and GenBank accession numbers of isolates included in the phylogenetic analyses.

## References

[1] Kazemzadeh C.M., Mohammadi H., Khodaparast S.A. (2019). Diversity and pathogenicity of *Botryosphaeriaceae* species on forest trees in the north of Iran. Eur. J. For. Res..

[2] Zhang W., Groenewald J.Z., Lombard L., Schumacher R.K., Phillips A.J.L., Crous P.W. (2021). Evaluating species in Botryosphaeriales. Persoonia.

[3] Heidari S., Gheisari M., Abastabar M., Pourabdollah M., Mirenayat M.S., Basharzad N., Seifi S., Tavakoli M., Jafarzadeh J., Ansari S. (2021). Genotyping and ın vitro antifungal susceptibility profile of *Neoscytalidium* species isolates from respiratory tract. Mycopathologia.

[4] Dolatabadi S., Nasirharandi S., Pourahmad M., Ahmadikia K., Mokhtari M., Najafzadeh M.J., Mohammadi R. (2022). Phaeohyphomycosis caused by *Neoscytalidium dimidiatum* in a COVID-19 patient. Rev. Iberoam. Micol..

[5] Chuang M.F., Ni H.F., Yang H.R., Shu S.L., Lai S.Y., Jiang Y.L. (2012). First report of stem canker disease of pitaya (*Hylocereus undatus* and *H*. *polyrhizus*) caused by *Neoscytalidium dimidiatum* in Taiwan. Plant Dis..

[6] Lan G.B., He Z.F., Xi P.G., Jiang Z.D. (2012). First report of brown spot disease caused by *Neoscytalidium dimidiatum* on *Hylocereus undatus* in Guangdong, Chinese Mainland. Plant Dis..

[7] Yi R.H., Gan L.J., Yan D.H., Wu Z.J., Tong Y.T., Wu F.F. (2013). Identification and biological characteristics of *Neoscytalidium dimidiatum* causing pitaya canker disease. Acta Phytophylacica Sin..

[8] Ezra D., Liarzi O., Gat T., Hershcovich M., Dudai M. (2013). First report of internal black rot caused by *Neoscytalidium dimidiatum* on *Hylocereus undatus* (pitahaya) fruit in Israel. Plant Dis..

[9] Mohd M.H., Salleh B., Zakaria L. (2013). Identification and molecular characterizations of *Neoscytalidium dimidiatum* causing stem canker of red-fleshed dragon fruit (*Hylocereus polyrhizus*) in Malaysia. J. Phytopathol..

[10] Sanahuja G., Lopez P., Palmateer A.J. (2016). First report of *Neoscytalidium dimidiatum* causing stem and fruit canker of *Hylocereus undatus* in Florida. Plant Dis..

[11] Hong C.F., Gazis R., Crane J.H., Zhang S. (2020). Prevalence and epidemics of Neoscytalidium stem and fruit canker on pitahaya (*Hylocereus* spp.) in South Florida. Plant Dis..

[12] Xu M., Peng Y., Qi Z., Yan Z., Yang L., He M.D., Li Q.X., Liu C.L., Ruan Y.Z., Wei S.S. (2018). Identification of *Neoscytalidium dimidiatum* causing canker disease of pitaya in Hainan, China. Australas. Plant Pathol..

[13] Balendres M.A., Taguiam J.D., Evallo E., Estigoy J., Cortaga C. (2022). Fruit Brown Rot Caused by Neoscytalidium dimidiatum on Selenicereus monacanthus in the Philippines.

[14] Serrato-Diaz L.M., Goenaga R. (2021). First report of *Neoscytalidium dimidiatum* causing stem canker on dragon fruit (*Hylocereus* spp.) in Puerto Rico. Plant Dis..

[15] Dy K.S., Wonglom P., Pornsuriya C., Sunpapao A. (2022). Morphological, molecular ıdentification and pathogenicity of *Neoscytalidium dimidiatum* causing stem canker of *Hylocereus polyrhizus* in Southern Thailand. Plants.

[16] Salunkhe V.N., Bhagat Y.S., Chavan S.B., Lonkar S.G., Kakade V.D. (2023). First report of *Neoscytalidium dimidiatum* causing stem canker of dragon fruit (*Hylocereus* spp.) in India. Plant Dis..

[17] Khoo Y.W., Tan H.T., Khaw Y.S., Li S.F., Chong K.P. (2023). First report of *Neoscytalidium dimidiatum* causing stem canker on *Selenicereus megalanthus* in Malaysia. Plant Dis..

[18] Espinoza-Lozano L., Sumba M., Calero A., Jiménez M.I., Quito-Avila D.F. (2023). First report of *Neoscytalidium dimidiatum* causing stem canker on yellow dragon fruit (*Hylocereus megalantus*) in Ecuador. Plant Dis..

[19] Polizzi G., Aiello D., Vitale A., Giuffrida F., Groenewald J.Z., Crous P.W. (2009). First Report of shoot blight, canker, and gummosis caused by *Neoscytalidium dimidiatum* on citrus in Italy. Plant Dis..

[20] Mayorquin J.S., Wang D.H., Twizeyimana M., Eskalen A. (2016). Identification, distribution, and pathogenicity of Diatrypaceae and Botryosphaeriaceae associated with citrus branch canker in the Southern California desert. Plant Dis..

[21] Al-Saadoon A.H., Ameen M.K.M., Hameed M.A., Al-Badran A., Ali Z. (2012). First report of grapevine dieback caused by *Lasiodiplodia theobromae* and *Neoscytalidium dimidiatum* in Basrah, Southern Iraq. Afr. J. Biotechnol..

[22] Rolshausen P.E., Akgul D.S., Perez R., Eskalen A., Gispert C. (2013). First report of wood canker caused by *Neoscytalidium dimidiatum* on grapevine in California. Plant Dis..

[23] Correia K.C., Silva M.A., Netto M.S.B., Vieira W.A.S., Camara M.P.S., Michereff S.J. (2016). First report of grapevine dieback caused by *Neoscytalidium hyalinum* in Brazil. Plant Dis..

[24] Oksal E., Çelik Y., Özer G. (2019). *Neoscytalidium dimidiatum* causes canker and dieback on grapevine in Turkey. Australas. Plant Dis. Notes.

[25] Akgül D.S., Savaş N.G., Özarslandan M. (2019). First report of wood canker caused by *Lasiodiplodia exigua* and *Neoscytalidium novaehollandiae* on grapevine in Turkey. Plant Dis..

[26] Bahmani Z., Abdollahzadeh J., Amini J., Evidente A. (2021). *Biscogniauxia rosacearum* the charcoal canker agent as a pathogen associated with grapevine trunk diseases in Zagros region of Iran. Sci. Rep..

[27] Türkölmez Ş., Derviş S., Çiftçi O., Dikilitas M. (2019). First report of *Neoscytalidium dimidiatum* causing shoot and needle blight of pines (*Pinus* spp.) in Turkey. Plant Dis..

[28] Alizadeh M., Safaie N., Shams-Bakhsh M., Mehrabadi M. (2022). *Neoscytalidium novaehollandiae* causes dieback on *Pinus eldarica* and its potential for infection of urban forest trees. Sci. Rep..

[29] Hajlaoui M.R., Nouri M.T., Hamrouni N., Trouillas F.P., Ben Yahmed N., Eddouzi J., Mnari-Hattab M. (2018). First record of dieback and decline of plum caused by *Neoscytalidium dimidiatum* in Tunisia. New Dis. Rep..

[30] Nouri M.T., Lawrence D.P., Yaghmour M.A., Michailides T.J., Trouillas F.P. (2018). *Neoscytalidium dimidiatum* causing canker, shoot blight and fruit rot of almond in California. Plant Dis..

[31] Oksal E., Yiğit T., Özer G. (2020). First report of *Neoscytalidium dimidiatum* causing shoot blight, dieback and canker of apricot in Turkey. J. Plant Pathol..

[32] Ören E., Koca G., Gencer R., Bayraktar H. (2020). First report of *Neoscytalidium novaehollandiae* associated with stem canker and branch dieback of almond trees. Australas. Plant Dis. Notes.

[33] Holland L.A., Trouillas F.P., Nouri M.T., Lawrence D.P., Crespo M., Doll D.A., Duncan R.A., Holtz B.A., Culumber C.M., Yaghmour M.A. (2021). Fungal pathogens associated with canker diseases of almond in California. Plant Dis..

[34] Elshafie A.E., Ba-Omar T. (2002). First report of *Albizia lebbeck* dieback caused by *Scytalidium dimidiatum* in Oman. Mycopathologia.

[35] Ray J.D., Burgess T., Lanoiselet V.M. (2010). First record of *Neoscytalidium dimidiatum* and *N*. *novaehollandiae* on *Mangifera indica* and *N*. *dimidiatum* on *Ficus carica* in Australia. Australas. Plant Dis. Notes.

[36] Fernández-Herrera E., Moreno-Salazar S.F., Rentería-Martínez M.E., Arratia-Castro A.A., Villar-Luna E. (2017). *Neoscytalidium dimidiatum*: Causal agent of dieback in *Ficus benjamina* L. in Mexico. Rev. Chapingo Ser. Hortic..

[37] Al-Bedak O.A., Mohamed R.A., Seddek N.H. (2018). First detection of *Neoscytalidium dimidiatum* associated with canker disease in Egyptian *Ficus* trees. For. Pathol..

[38] Güney İ.G., Bozoğlu T., Özer G., Türkölmez Ş., Derviş S. (2022). First report of *Neoscytalidium dimidiatum* associated with dieback and canker of common fig (*Ficus carica* L.) in Turkey. J. Plant Dis. Prot..

[39] Yeganeh S., Mohammadi H. (2022). Sooty canker, a destructive disease of banyan (*Ficus benghalensis* L.) trees in landscapes of Kish Island (Iran). Urban For. Urban Green..

[40] Ahmadpour S.A., Mehrabi-Koushki M., Farokhinejad R., Mirsoleymani Z. (2023). Characterization and pathogenicity of *Neoscytalidium novaehollandiae* causing dieback and sooty canker in Iran. Trop. Plant Pathol..

[41] Gusella G., Fiore G., Vitale A., Felts D.G., Michailides T.J. (2023). New findings on the effects of different factors involved in fig limb dieback caused by *Neoscytalidium dimidiatum* in California. Eur. J. Plant Pathol..

[42] Derviş S., Türkölmez Ş., Çiftçi O., Ulubaş Serçe Ç., Dikilitas M. (2019). First report of *Neoscytalidium dimidiatum* causing canker, shoot blight, and root rot of pistachio in Turkey. Plant Dis..

[43] Hashemi H., Mohammadi H. (2016). Identification and characterization of fungi associated with internal wood lesions and decline disease of willow and poplar trees in Iran. For. Pathol..

[44] Türkölmez Ş., Derviş S., Çiftçi O., Ulubaş Serçe Ç., Türkölmez C.G., Dikilitas M. (2019). First report of *Neoscytalidium dimidiatum* causing dieback, shoot blight, and branch canker of willow trees in Turkey. Plant Dis..

[45] Fullerton R.A., Sutherland P.A., Rebstock R.S., Hieu N.T., Thu N.N.A., Linh D.T., Thanh N.T.K., Van Hoa N. The life cycle of dragon fruit canker caused by *Neoscytalidium dimidiatum* and implications for control. Proceedings of the Dragon Fruit Regional Network Initiation Workshop.

[46] Al-Sadi A.M., Al-Ghaithi A.G., Al-Fahdi N., Al-Yahyai R. (2014). Characterization and pathogenicity of fungal pathogens associated with root diseases of citrus in Oman. Int. J. Agric. Biol..

[47] Türkölmez Ş., Derviş S., Çiftçi O., Serçe Ç.U., Dikilitas M. (2019). New disease caused by *Neoscytalidium dimidiatum* devastates tomatoes (*Solanum lycopersicum*) in Turkey. Crop Prot..

[48] Jiménez Luna I., Doll D., Ashworth V., Trouillas F.P., Rolshausen P.E. (2022). Comparative profiling of wood canker pathogens from spore traps and symptomatic plant samples within California almond and walnut orchards. Plant Dis..

[49] Güney İ.G., Bozoğlu T., Özer G., Derviş S. (2023). A novel blight and root rot of chickpea: A new host for *Neoscytalidium dimidiatum*. Crop Prot..

[50] Crous P.W., Slippers B., Wingfield M.J., Rheeder J., Marasas W.F., Philips A.J., Alves A., Burgess T., Barber P., Groenewald J.Z. (2006). Phylogenetic lineages in the Botryosphaeriaceae. Stud. Mycol..

[51] Pavlic D., Wingfield M.J., Barber P., Slippers B., Hardy G.E., Burgess T.I. (2008). Seven new species of the Botryosphaeriaceae from baobab and other native trees in Western Australia. Mycologia.

[52] Phillips A.J., Alves A., Abdollahzadeh J., Slippers B., Wingfield M.J., Groenewald J.Z., Crous P.W. (2013). The Botryosphaeriaceae: Genera and species known from culture. Stud. Mycol..

[53] Huang S.K., Tangthirasunun N., Phillips A.J., Dai D.Q., Wanasinghe D.N., Wen T.C., Bahkali A.H., Hyde K.D., Kang J.C. (2016). Morphology and phylogeny of *Neoscytalidium orchidacearum* sp. nov. (Botryosphaeriaceae). Mycobiology.

[54] Calvillo-Medina R.P., Martinez-Neria M., Mena-Portales J., Barba-Escoto L., Raymundo T., Campos-Guillen J., Jones G.H., Reyes-Grajeda J.P., Gonzalez Y.M.J.A., Bautista-de Lucio V.M. (2019). Identification and biofilm development by a new fungal keratitis aetiologic agent. Mycoses.

[55] Wonglom P., Pornsuriya C., Sunpapao A. (2023). A new species of *Neoscytalidium hylocereum* sp. nov. causing canker on red-fleshed dragon fruit (*Hylocereus polyrhizus*) in Southern Thailand. J. Fungi.

[56] Nattrass R.M. (1933). A new species of *Hendersonula* (*H. toruloidea*) on deciduous trees in Egypt. Trans. Br. Mycol. Soc..

[57] Sutton B.C., Dyko B.J. (1989). Revision of *Hendersonula*. Mycol. Res..

[58] Farr D.F., Elliott M., Rossman A.Y., Edmonds R.L. (2005). *Fusicoccum arbuti* sp. nov. causing cankers on Pacific madrone in western North America with notes on *Fusicoccum dimidiatum*, the correct name for *Scytalidium dimidiatum* and *Natrassia mangiferae*. Mycologia.

[59] Penzig O. (1882). Funghi agrumicoli. Contribuzione allo studio dei funghi parassiti degli agrumi. Michelia.

[60] Wilson E.E. (1947). The branch wilt of Persian walnut trees and its cause. Hilgardia.

[61] Campbell C.K., Mulder J.L. (1977). Skin and nail infection by *Scytalidium hyalinum* sp. nov. Sabouraudia.

[62] Slippers B., Johnson G.I., Crous P.W., Coutinho T.A., Wingfield B.D., Wingfield M.J. (2005). Phylogenetic and morphological re-evaluation of the Botryosphaeria species causing diseases of *Mangifera indica*. Mycologia.

[63] Sydow H., Sydow P., Butler E.J. (1916). Fungi Indiae Orientalis. V. Ann. Mycol..

[64] Madrid H., Ruiz-Cendoya M., Cano J., Stchigel A., Orofino R., Guarro J. (2009). Genotyping and in vitro antifungal susceptibility of *Neoscytalidium dimidiatum* isolates from different origins. Int. J. Antimicrob. Agents.

[65] Xu J. (2020). Fungal species concepts in the genomics era. Genome.

[66] Maharachchikumbura S.S.N., Chen Y., Ariyawansa H.A., Hyde K.D., Haelewaters D., Perera R.H., Samarakoon M.C., Wanasinghe D.N., Bustamante D.E., Liu J. (2021). Integrative approaches for species delimitation in Ascomycota. Fungal Divers..

[67] Katoh K., Rozewicki J., Yamada K.D. (2019). MAFFT online service: Multiple sequence alignment, interactive sequence choice and visualization. Brief. Bioinform..

[68] Kumar S., Stecher G., Li M., Knyaz C., Tamura K. (2018). MEGA X: Molecular evolutionary genetics analysis across computing platforms. Mol. Biol. Evol..

[69] Stamatakis A. (2014). RAxML version 8: A tool for phylogenetic analysis and post-analysis of large phylogenies. Bioinformatics.

[70] Swofford D.L. (2003). PAUP*: Phylogenetic Analysis Using Parsimony. (* and Other Methods).

[71] Ronquist F., Teslenko M., Van Der Mark P., Ayres D.L., Darling A., Höhna S., Huelsenbeck J.P. (2012). MrBayes 3.2: Efficient Bayesian phylogenetic inference and model choice across a large model space. Syst. Biol..

[72] Coutinho I.B.L., Cardoso J.E., Lima C.S., Lima J.S., Goncalves F.J.T., Silva A.M.S., Freire F.C.O. (2018). An emended description of *Neofusicoccum brasiliense* and characterization of *Neoscytalidium* and *Pseudofusicoccum* species associated with tropical fruit plants in northeastern Brazil. Phytotaxa.

[73] Hohenfeld C.S., Santana M.P., Junior L.R.C., de Oliveira E.J., de Oliveira S.A.S. (2018). Modelling growth characteristics and aggressiveness of *Neoscytalidium hyalinum* and *Fusarium solani* associated with black and dry root rot diseases on cassava. Trop. Plant Pathol..

[74] Sabernasab M., Jamali S., Marefat A., Abbasi S. (2019). Morphological and molecular characterization of *Neoscytalidium novaehollandiae*, the cause of *Quercus brantii* dieback in Iran. Phytopathol. Mediterr..

[75] Gusella G., Morgan D.P., Michailides T.J. (2021). Further ınvestigation on limb dieback of fig (*Ficus carica*) Caused by *Neoscytalidium dimidiatum* in California. Plant Dis..

[76] Güney İ.G., Özer G., Türkölmez Ş., Derviş S. (2022). Canker and leaf scorch on olive (*Olea europaea* L.) caused by *Neoscytalidium dimidiatum* in Turkey. Crop Prot..

[77] Goudarzi A., Moslehi M. (2020). Distribution of a devastating fungal pathogen in mangrove forests of southern Iran. Crop Prot..

[78] Nurul Nadiah M.A., Mohamed Nor N.M.I., Latiffah Z., Masratul Hawa M. (2017). First report of leaf blight on white spider lily caused by *Neoscytalidium dimidiatum* in Malaysia. New Dis. Rep..

[79] Zaeimian Z., Fotouhifar K.B. (2023). First report of *Neoscytalidium dimidiatum* as the causal agent of leaf blight on *Clivia miniata*. Sci. Rep..

[80] Pandey R.S., Bhargava S.N., Shukla D.N., Khati D.V.S. (1981). A new leaf spot disease of mango. Plant Dis..

[81] Reckhaus P., Adamous I. (1987). Hendersonula dieback of mango in Niger. Plant Dis..

[82] Mendes M.A.S., da Silva V.L., Dianese J.C., Ferreira M.A.S.V., dos Santos C.E.N., Urben A.F., Castro C., Gomes Neto E. (1998). Fungos em Plants no Brasil.

[83] Sakalidis M.L., Ray J.D., Lanoiselet V., St J., Hardy G.E., Burgess T.I. (2011). Pathogenic Botryosphaeriaceae associated with *Mangifera indica* in the Kimberley Region of Western Australia. Eur. J. Plant Pathol..

[84] Marques M.W., Lima N.B., de Morais M.A., Michereff S.J., Phillips A.J., Câmara M.P. (2013). *Botryosphaeria*, *Neofusicoccum*, *Neoscytalidium* and *Pseudofusicoccum* species associated with mango in Brazil. Fungal Divers..

[85] Dolatabad H.K., Javan-Nikkhah M., Shier W.T. (2017). Evaluation of antifungal, phosphate solubilisation, and siderophore and chitinase release activities of endophytic fungi from *Pistacia vera*. Mycol. Prog..

[86] Kurt Ş., Uysal A., Soylu E.M., Kara M., Soylu S. (2019). First record of *Neoscytalidium novaehollandiae* associated with pistachio dieback in the Southeastern Anatolia region of Turkey. Mycol. Iran.

[87] Williams L., Hayne S.C. (1982). Index of Plant Diseases in West Virginia.

[88] Farr D.F., Bills G.F., Chamuris G.P., Rossman A.Y. (1989). Fungi on Plants and Plant Products in the United States.

[89] Mathur R.S. (1979). The Coelomycetes of India.

[90] Gusella G., Di Pietro C., Vecchio L., Campo G., Polizzi G. (2023). Branch canker and dieback of *Meryta denhamii* caused by *Neofusicoccum parvum* and *Neoscytalidium dimidiatum* in Italy. Australas. Plant Dis. Notes.

[91] Peregrine W.T.H., Ahmad K.B. (1982). Brunei: A first annotated list of plant diseases and associated organisms. Phytopathol. Pap..

[92] Kranz J. (1963). Fungi collected in the Republic of Guinea, Collections from the Kindia area in 1962. Sydowia.

[93] Xie H.H., Long L., Huang S., Mao L., Huang Q., Wang L., Li J. (2021). First Report of Black Spot Caused by *Neoscytalidium dimidiatum* on Sisal in Guangxi, China. Plant Dis..

[94] Johnston A. (1960). A supplement to a host list of plant diseases in Malaya. Mycol. Pap..

[95] Kee Y.J., Suhaimi N.N., Zakaria L., Mohd M.H. (2017). Characterisation of *Neoscytalidium dimidiatum* causing leaf blight on *Sansevieria trifasciata* in Malaysia. Australas. Plant Dis. Notes.

[96] Monteles R.P., Sousa E.S., da Silva Matos K., de Brito V.S.T., de Melo M.P., Beserra J.E.A. (2020). *Neoscytalidium dimidiatum* causes leaf blight on *Sansevieria trifasciata* in Brazil. Australas. Plant Dis. Notes.

[97] Crous P.W., Hernández-Restrepo M., Schumacher R.K., Cowan D.A., Maggs-Kölling G., Marais E., Groenewald J.Z. (2021). New and interesting fungi. Fungal Syst. Evol..

[98] Esmaeili N., Mohammadi H., Sohrabi M. (2023). Barberry (*Berberis vulgaris* L.) as an alternative host of grapevine fungal trunk pathogens. Eur. J. Plant Pathol..

[99] Ebbels D.L., Allen D.J. (1979). A supplementary and annotated list of plant diseases, pathogens and associated fungi in Tanzania. Phytopathol. Pap..

[100] Kuruppu M., Siddiqui Y., Ahmad K., Ali A. (2021). First report of postharvest stem end rot disease on MD2 pineapple fruits caused by *Neoscytalidium dimidiatum* in Malaysia. Plant Dis..

[101] Feijo F.M., Silva M.J.S., Nascimento A.D., Infante N.B., Ramos-Sobrinho R., Assuncao I.P., Lima G.S.A. (2019). Botryosphaeriaceae species associated with the pickly pear cactus, *Nopalea cochenillifera*. Trop. Plant Pathol..

[102] Yi R.H., Lin Q.L., Mo J.J., Wu F.F., Chen J. (2015). Fruit internal brown rot caused by *Neoscytalidium dimidiatum* on pitahaya in Guangdong Province, China. Australas. Plant Dis. Notes.

[103] Boa E., Lenné J. (1994). Diseases of Nitrogen Fixing Trees in Developing Countries. An Annotated List.

[104] Watson A.J. (1971). Foreign Bacterial and Fungus Diseases of Food, Forage, and Fiber Crops.

[105] de Mello J.F., Brito A.C.Q., Motta C.M.S., Vieira J.C.B., Michereff S.J., Machado A.R. (2019). First report of *Neoscytalidium dimidiatum* causing root rot in sweet potato in Brazil. Plant Dis..

[106] de Mello J.F., Brito A.C.Q., Vieira J.C.B., Camara M.P.S., Michereff S.J., de Souza-Motta C.M., Machado A.R. (2021). Identification and pathogenicity of Botryosphaeriaceae species associated with root and stem rot of sweet potato in Brazil. Plant Pathol..

[107] Mirtalebi M., Sabahi F., Banihashemi Z. (2019). Fruit rot caused by *Neoscytalidium hyalinum* on melon in Iran. Australas. Plant Dis. Notes.

[108] California Fungi. Nos. 1-1325. Exsiccati Set 1931–1970, N/A Pages. https://data.nal.usda.gov/dataset/united-states-national-fungus-collections-fungus-host-dataset.

[109] Lin C.H., Chen Y.X., Liu W.B., Wu W.Q., Miao W.G., Zheng F.C. (2017). First report of *Dioscorea esculenta* dieback caused by *Neoscytalidium dimidiatum* in China. Plant Dis..

[110] Arrieta-Guerra J.J., Díaz-Cabadiaz A.T., Pérez-Pazos J.V., Cadena-Torres J., Sánchez-López D.B. (2021). Fungi associated with dry rot disease of yam (*Dioscorea rotundata* Poir.) tubers in Cordoba, Colombia. Agron. Mesoam..

[111] Ören E., Koca G., Bayraktar H. (2020). First report of *Neoscytalidium novaehollandiae* associated with branch dieback on Japanese persimmon in Turkey. J. Plant Pathol..

[112] Davison A.D. (1972). Factors affecting development of madrone canker. Plant Dis. Rep..

[113] Shaw C.G. (1973). Host Fungus Index for the Pacific Northwest-I. Hosts.

[114] Ginns J.H. (1986). Compendium of plant disease and decay fungi in Canada 1960–1980. Res. Br. Can. Agric. Publ..

[115] French A.M. (1989). California Plant Disease Host Index.

[116] Tsahouridou P.C., Thanassoulopoulos C.C. (2000). First report of *Hendersonula toruloidea* as a foliar pathogen of strawberry-tree (*Arbutus unedo*) in Europe. Plant Dis..

[117] Machado A.R., Pinho D.B., Dutra D.C., Pereira O.L. (2012). First report of collar and root rot of physic nut (*Jatropha curcas*) caused by *Neoscytalidium dimidiatum* in Brazil. Plant Dis..

[118] Machado A.R., Pinho D.B., Pereira O.L. (2014). Phylogeny, identification and pathogenicity of the Botryosphaeriaceae associated with collar and root rot of the biofuel plant *Jatropha curcas* in Brazil, with a description of new species of *Lasiodiplodia*. Fungal Divers..

[119] Hughes S.J. (1952). Fungi From the Gold Coast. I. Mycol. Pap..

[120] Nattrass R.M. (1961). Host lists of Kenya fungi and bacteria. Mycol. Pap..

[121] Machado A.R., Pinho D.B., Oliveira S.A.S., Pereira O.L. (2014). New occurrences of Botryosphaeriaceae causing black root rot of cassava in Brazil. Trop. Plant Pathol..

[122] Brito A.C.Q., de Mello J.F., Camara M.P.S., Vieira J.C.B., Michereff S.J., Souza-Motta C.M., Machado A.R. (2020). Diversity and pathogenicity of Botryosphaeriaceae species associated with black root rot and stem cutting dry rot in *Manihot esculenta* in Brazil. Eur. J. Plant Pathol..

[123] Sangpueak R., Duchanee S., Saengchan C., Papathoti N.K., Hoang N.H., Thanh T.L., Phansak P., Buensanteai N. (2023). Identification of cassava black stem and root rot agents in Thailand. Chil. J. Agric. Res..

[124] Pande A., Rao V.G. (1998). A Compendium Fungi on Legumes from India.

[125] Chandra S. (1974). Some new leaf-spot diseases from Allahabad (India). Beih. Nova Hedwig..

[126] Sakalidis M.L., Hardy G.E.S., Burgess T.I. (2011). Endophytes as potential pathogens of the baobab species *Adansonia gregorii*: A focus on the Botryosphaeriaceae. Fungal Ecol..

[127] Al Raish S.M., Saeed E.E., Sham A., Alblooshi K., El-Tarabily K.A., AbuQamar S.F. (2020). Molecular characterization and disease control of stem canker on royal poinciana (*Delonix regia*) caused by *Neoscytalidium dimidiatum* in the United Arab Emirates. Int. J. Mol. Sci..

[128] Galea V.J. (2021). Use of stem ımplanted bioherbicide capsules to manage an ınfestation of *Parkinsonia aculeata* in Northern Australia. Plants.

[129] (1960). Index of Plant Diseases in the United States. U.S.D.A. Agriculture Handbook.

[130] Alidadi A., Kowsari M., Javan-Nikkhah M., Jouzani G.R.S., Rastaghi M.E. (2019). New pathogenic and endophytic fungal species associated with Persian oak in Iran. Eur. J. Plant Pathol..

[131] French A.M. (1987). California Plant Disease Host Index. Part 1: Fruit and Nuts.

[132] Wilson E.E. (1949). The pycnidial stage of the walnut branch wilt fungus, *Exosporina fawcettii*. Phytopathology.

[133] Sommer N.F. (1955). Sunburn predisposes walnut trees to branch-wilt. Phytopathology.

[134] Phillips A.J.L., Oudemans P.V., Correia A., Alves A. (2006). Characterisation and epitypification of *Botryosphaeria corticis*, the cause of blueberry cane canker. Fung Divers..

[135] Chen S.F., Fichtner E., Morgan D.P., Michailides T.J. (2013). First report of *Lasiodiplodia citricola* and *Neoscytalidium dimidiatum* causing death of graft union of English walnut in California. Plant Dis..

[136] Chen S., Morgan D.P., Hasey J.K., Anderson K., Michailides T.J. (2014). Phylogeny, morphology, distribution, and pathogenicity of Botryosphaeriaceae and Diaporthaceae from English walnut in California. Plant Dis..

[137] Derviş S., Türkölmez Ş., Çiftçi O., Ulubaş Serçe Ç., Dikilitas M. (2019). First report of *Neoscytalidium dimidiatum* causing black canker and root rot of walnut in Turkey. Plant Dis..

[138] Çiftçi O., Özer G., Türkölmez Ş., Derviş S. (2023). *Lasiodiplodia theobromae* and *Neoscytalidium dimidiatum* associated with grafted walnut (*Juglans regia* L.) decline in Turkey. J. Plant Dis. Prot..

[139] Bagherabadi S., Zafari D., Maharachchikumbura S.S. (2022). *Neoscytalidium dimidiatum* as one of the fungal agents associated with walnut decline in Iran. J. Nuts.

[140] Güney İ.G., Özer G., Turan İ., Koşar İ., Derviş S. (2021). First report of *Neoscytalidium dimidiatum* causing foliar and stem blight of lavender in Turkey. J. Plant Pathol..

[141] Özer G., Günen T.U., Koşar İ., Güler İ.G., Derviş S. (2022). First report of *Neoscytalidium dimidiatum* causing blight of *Melissa officinalis* in Turkey. J. Plant Dis. Prot..

[142] Alkan M., Özer G., Koşar I., Güney I.G., Derviş S. (2022). First report of leaf blight of Turkish oregano (*Origanum onites*) caused by *Neoscytalidium dimidiatum* in Turkey. J. Plant Pathol..

[143] Derviş S., Güney İ.G., Koşar İ., Bozoğlu T., Özer G. (2021). First report of *Neoscytalidium novaehollandiae* on common sage (*Salvia officinalis*). Australas. Plant Dis..

[144] Kadkhoda-Hematabadi S., Mohammadi H., Sohrabi M. (2023). Morphological and molecular identification of plant pathogenic fungi associated with necrotic wood tissues of pomegranate trees in Iran. J. Plant Pathol..

[145] Ghasemi-Sardareh R., Mohammadi H. (2020). Characterization and pathogenicity of fungal trunk pathogens associated with declining of neem (*Azadirachta indica* A. Juss) trees in Iran. J. Plant Pathol..

[146] Ahmad S., Iqbal S.H., Khalid A.N. (1997). Fungi of Pakistan. Sultan Ahmad Mycological Society of Pakistan.

[147] Abdulrahman D.N., Haleem R.A. (2023). Morphological and molecular characterization of *Neoscytalidium* isolates that cause canker and dieback in eucalyptus and chinaberry trees in Iraq. Plant Prot. Sci..

[148] Georghiou G.P., Papadopoulos C. (1957). A Second List of Cyprus Fungi.

[149] Paxton J.D., Wilson E.E., Davis J.R. (1964). Branch wilt disease of fig caused by *Hendersonula toruloidea*. Plant Dis. Rep..

[150] Oksal E. (2022). Prevalence, molecular characterization, and variety reactions of *Neoscytalidium novaehollandiae* on mulberries in Turkey. Not. Bot. Horti Agrobot. Cluj-Napoca.

[151] Meredith D.S. (1963). Tip rot of banana fruits in Jamaica. Trans. Br. Mycol. Soc..

[152] Meredith D.S. (1969). Fungal diseases of bananas in Hawaii. Plant Dis. Rep..

[153] Raabe R.D., Conners I.L., Martinez A.P. (1981). Checklist of Plant Diseases in Hawaii.

[154] Al-Tememe Z.A.M., Lahuf A., Abdalmoohsin R.G., Al-Amirry A.T. (2019). Occurrence, identification, pathogenicity and control of *Neoscytalidium dimidiatum* fungus, the causal agent of sooty canker on *Eucalyptus camaldulensis* in Kerbala Province of Iraq. Plant Arch..

[155] Ismail S.I., Ahmad Dahlan K., Abdullah S., Zulperi D. (2021). First Report of *Neoscytalidium dimidiatum* causing fruit rot on guava (*Psidium guajava* L.) in Malaysia. Plant Dis..

[156] Panahandeh S., Mohammadi H., Gramaje D. (2019). Trunk disease fungi associated with *Syzygium cumini* in Iran. Plant Dis..

[157] Williams T.H., Liu P.S.W. (1976). A host list of plant diseases in Sabah, Malaysia. Phytopathol. Pap..

[158] Suwannarach N., Kumla J., Lumyong S. (2018). Leaf spot on cattleya orchid caused by *Neoscytalidium orchidacearum* in Thailand. Can. J. Plant Pathol..

[159] Chang C.W., Chen C.Y., Wang C.L., Chung W.H. (2020). First report of leaf blight on *Cattleya* × hybrid caused by *Neoscytalidium dimidiatum* in Taiwan. J. Plant Pathol..

[160] Derviş S., Türkölmez Ş., Güney İ.G., Alkan M., Ozer G. (2023). First report of needle blight of blue spruce (*Picea pungens*) caused by *Neoscytalidium dimidiatum* in Turkey. J. Plant Pathol..

[161] Nourian A., Salehi M., Safaie N., Khelghatibana F., Abdollahzadeh J. (2021). Fungal canker agents in apple production hubs of Iran. Sci. Rep..

[162] Sha S., Wang Z., Hao H., Wang L., Feng H. (2022). First report of *Neoscytalidium dimidiatum* inducing canker disease on apple trees in China. J. Plant Pathol..

[163] Ören E., Palacıoğlu G., Ozan G.N., Çelik K., Bayraktar H. (2022). First report of *Neoscytalidium novaehollandiae* associated with canker and branch dieback on cherry trees in Turkey. J. Plant Pathol..

[164] Ören E., Palacıoğlu G., Ozan G.N., Bayraktar H. (2022). First report of *Neoscytalidium novaehollandiae* associated with branch dieback and stem cankers on plum in Turkey. J. Plant Pathol..

[165] English H., Davis J.R., Devay J.E. (1975). Relationship of *Botryosphaeria dothidea* and *Hendersonula toruloidea* to a canker disease of almond. Phytopathology.

[166] Burgess T.I., Barber P.A., St. J., Hardy G.E. (2005). *Botryosphaeria* spp. associated with eucalypts in Western Australia, including the description of *Fusicoccum macroclavatum* sp. nov.. Australas. Plant Pathol..

[167] Oksal E., Özer G. (2021). First report of shoot blight and branch canker of *Pyrus communis* by *Neoscytalidium novaehollandiae* in Turkey. J. Plant Pathol..

[168] Espargham N., Mohammadi H., Gramaje D. (2020). A Survey of Trunk Disease Pathogens within Citrus Trees in Iran. Plants.

[169] Alananbeh K.M., Al-Qasim M., Gharaibeh A., Al-Hiary H.A. (2020). First report of shoot blight caused by *Neoscytalidium dimidiatum* on citrus in Jordan. Plant Dis..

[170] Calavan E.C., Wallace J.M. (1954). *Hendersonula toruloidea* Nattrass on *Citrus* in California. Phytopathology.

[171] Adesemoye A.O., Mayorquin J.S., Wang D.H., Twizeyimana M., Lynch S.C., Eskalen A. (2014). Identification of species of Botryosphaeriaceae causing bot gummosis in citrus in California. Plant Dis..

[172] Doidge E.M. (1950). The South African fungi and lichens to the end of 1945. Bothalia.

[173] Ogawa J.M. (1954). The occurrence of *Hendersonula toruloidea* Nattrass on *Populus* species in California. Plant Dis. Rep..

[174] Derviş S., Özer G., Türkölmez Ş. (2020). First report of *Neoscytalidium novaehollandiae* causing stem blight on tomato in Turkey. J. Plant Pathol..

[175] Derviş S., Özer G., Türkölmez Ş. (2020). First report of *Neoscytalidium dimidiatum* causing tuber rot of potato in Turkey. J. Plant Pathol..

[176] Hashemi H., Mohammadi H., Abdollahzadeh J. (2017). Symptoms and fungi associated with elm trees decline in Iran. Eur. J. For. Res..

[177] Wangikar P.D., Raut J.G., Gopalkrishna N. (1969). Drying of grape vines caused by *Hendersonula toruloidea*. Indian Phytopathol..

[178] Sarbhoy A.K., Lal G., Varshney J.L. (1971). Fungi of India (1967–1971).

[179] Natour R.M., Ahmed J.M. (1969). Control of branch wilt disease of grape. Plant Dis. Rep..

[180] Ören E., Palacıoğlu G., Koca G., Ozan G.N., Bayraktar H. (2022). First report of *Neoscytalidium dimidiatum* causing branch dieback and canker on apple in Turkey. J. Plant Pathol..

[181] Xu M., Liu C.L., Fu Y., Liao Z.W., Guo P.Y., Xiong R., Cheng Y., Wei S.S., Huang J.Q., Tang H. (2020). Molecular characterization and expression analysis of pitaya (*Hylocereus polyrhizus*) HpLRR genes in response to *Neoscytalidium dimidiatum* infection. BMC Plant Biol..

[182] Garcia J.F., Lawrence D.P., Morales-Cruz A., Travadon R., Minio A., Hernandez-Martinez R., Rolshausen P.E., Baumgartner K., Cantu D. (2021). Phylogenomics of plant-associated Botryosphaeriaceae Species. Front. Microbiol..

[183] Türkölmez Ş., Eren A., Özer G., Derviş S. (2022). The effect of *Talaromyces funiculosus* ST976 isolated from pistacia vera rhizosphere on phosphorus solubility in soil samples with different physicochemical properties. J. Agric. Nat..

[184] Kuan C.S., Ng K.P., Yew S.M., Umar Meleh H., Seow H.F., How K.N., Yeo S.K., Jee J.M., Tan Y.C., Yee W.Y. (2023). Comparative genomic and phenotypic analyses of pathogenic fungi *Neoscytalidium dimidiatum* and *Bipolaris papendorfii* isolated from human skin scraping. Braz. J. Microbiol..

[185] Slippers B., Wingfield M.J. (2007). Botryosphaeriaceae as endophytes and latent pathogens of woody plants: Diversity, ecology and impact. Fungal Biol. Rev..

[186] Khan A.H. (1959). Some new diseases of Citrus in Pakistan. Mycopathol. Mycol. Appl..

[187] Paxton J.D., Wilson E.E. (1965). Anatomical and physiological aspects of branch wilt disease of Persian walnut. Phytopathology.

[188] Hassan W.A., Haleem R.A., Hassan P.H. (2011). Effect of heat-stress predisposition on the development of sooty canker caused by *Neoscytalidium dimidiatum* (Penz.) Crous and Slippers. Acta Agrobot..

[189] Zhu X.M., Liu X.F. (2012). A new species and genus distribution record from China: *Neoscytalidium novaehollandiae*. Indian. J. Microbiol..

[190] Corlett R.T. (2017). Frugivory and seed dispersal by vertebrates in tropical and subtropical Asia: An update. Glob. Ecol. Conserv..

[191] Belair M., Restrepo-Leal J.D., Praz C., Fontaine F., Remond C., Fernandez O., Besaury L. (2023). Botryosphaeriaceae gene machinery: Correlation between diversity and virulence. Fungal Biol..

[192] Nagel J.H., Wingfield M.J., Slippers B. (2021). Increased abundance of secreted hydrolytic enzymes and secondary metabolite gene clusters define the genomes of latent plant pathogens in the *Botryosphaeriaceae*. BMC Genom..

[193] Reveglia M., Masi A., Cimmino T., Cinelli L., Mugnai A. (2019). Evidente phytotoxins produced by *Lasiodiplodia laeliocattleyae* involved in Botryosphaeria dieback of grapevines in Brazil. Phytopathol Mediterr..

[194] Trotel-Aziz P., Robert-Siegwald G., Fernandez O., Leal C., Villaume S., Guise J.F., Abou-Mansour E., Lebrun M.H., Fontaine F. (2022). Diversity of *Neofusicoccum parvum* for the production of the phytotoxic metabolites (-)-terremutin and (R)-mellein. J. Fungi.

[195] Bénard-Gellon M., Farine S., Goddard M.L., Schmitt M., Stempien E., Pensec F., Laloue H., Mazet-Kieffer F., Fontaine F., Larignon P. (2015). Toxicity of extracellular proteins from *Diplodia seriata* and *Neofusicoccum parvum* involved in grapevine Botryosphaeria dieback. Protoplasma.

[196] Cimmino A., Cinelli T., Evidente M., Masi M., Mugnai L., Silva M.A., Michereff S.J., Surico G., Evidente A. (2016). Phytotoxic fungal exopolysaccharides produced by fungi involved in grapevine trunk diseases. Nat. Prod. Commun..

[197] Andolfi A., Maddau L., Cimmino A., Linaldeddu B.T., Basso S., Deidda A., Serra S., Evidente A. (2014). Lasiojasmonates A–C, three jasmonic acid esters produced by *Lasiodiplodia* sp., a grapevine pathogen. Phytochemistry.

[198] Yu C., Diao Y., Lu Q., Zhao J., Cui S., Xiong X., Lu A., Zhang X., Liu H. (2022). Comparative genomics reveals evolutionary traits, mating strategies, and pathogenicity-related genes variation of Botryosphaeriaceae. Front. Microbiol..

[199] Drula E., Garron M.L., Dogan S., Lombard V., Henrissat B., Terrapon N. (2022). The carbohydrate-active enzyme database: Functions and literature. Nucleic Acids Res..

[200] Xu M., Liu C.L., Luo J., Qi Z., Yan Z., Fu Y., Wei S.S., Tang H. (2019). Transcriptomic de novo analysis of pitaya (*Hylocereus polyrhizus*) canker disease caused by *Neoscytalidium dimidiatum*. BMC Genom..

[201] Wang M., Wang Z., Wei S., Xie J., Huang J., Li D., Hu W., Li H., Tang H. (2022). Identification of RT-qPCR reference genes suitable for gene function studies in the pitaya canker disease pathogen *Neoscytalidium dimidiatum*. Sci. Rep..

[202] Arkam M., Alves A., Lopes A., Cechova J., Pokluda R., Eichmeier A., Zitouni A., Mahamedi A.E., Berraf-Tebbal A. (2021). Diversity of Botryosphaeriaceae causing grapevine trunk diseases and their spatial distribution under different climatic conditions in Algeria. Eur. J. Plant Pathol..

[203] Xian X., Lin S., Zhu G., Wei X., Qin W., Zhong Y. (2018). Indoor virulence and field effects of fungicides on pitaya canker. J. South. Agric..

[204] Noegrahati S., Sulasmi S., Hemadi E., Asviastuti S. (2019). Dissipation pattern of azoxystrobin and difenoconazole in red dragon fruit (*Hylocereus polyrhizus*) cultivated in Indonesian highland (West Java) and coastal area (D.I. Jogyakarta) and its implication for dietary risk assessment. Food Qual. Saf..

[205] Eraslan Sür A., Oksal E. (2021). In vitro efficiency of some fungicides against *Neoscytalidium dimidiatum* (Penz.) Crous and Slippers causing sudden shoot dry on apricot trees. Turk. J. Agric.-Food Sci. Technol..

[206] Riska J., Budiyanti T., Husada E.D., Indriyani N.L.P., Hadiati S., Muas I., Mansyah E. (2023). Stem canker of dragon fruit (*Hylocereus polyrhizus*): *Neoscytalidium* sp. is a pathogen of the disease and its control using sodium salt. Plant Protect. Sci..

[207] Campos-Rivero G., Sánchez-Teyer L.F., De la Cruz-Arguijo E.A., Ramírez-González M.S., Larralde-Corona C.P., Narváez-Zapata J.A. (2019). Bioprospecting for fungi with potential pathogenic activity on leaves of *Agave tequilana* Weber var. Azul.. J. Phytopathol..

[208] Taguiam J.D., Evallo E., Bengoa J., Maghirang R., Balendres M.A. (2020). Susceptibility of the three dragon fruit species to stem canker and growth inhibition of *Neoscytalidium dimidiatum* by chemicals. J. Plant Pathol..

[209] Ratanaprom S., Nakkanong K., Nualsri C., Jiwanit P., Rongsawat T., Woraathakorn N. (2021). Overcoming encouragement of dragon fruit plant (*Hylocereus undatus*) against stem brown spot disease caused by *Neoscytalidium dimidiatum* using *Bacillus subtilis* combined with sodium bicarbonate. Plant Pathol. J..

[210] Wang F., Zhang R., Yuan Z., Chen P. (2021). Biological prevention and control of pitaya fruit canker disease using endophytic fungi isolated from papaya. Arch. Microbiol..

[211] Al Hamad B.M., Al Raish S.M., Ramadan G.A., Saeed E.E., Alameri S.S.A., Al Senaani S.S., AbuQamar S.F., El-Tarabily K.A. (2021). Effectiveness of augmentative biological control of *Streptomyces griseorubens* UAE2 depends on 1-aminocyclopropane-1-carboxylic acid deaminase activity against *Neoscytalidium dimidiatum*. J. Fungi.

[212] Lin S., Chen X., Xie L., Zhang Y., Zeng F., Long Y., Ren L., Qi X., Wei J. (2023). Biocontrol potential of lipopeptides produced by *Paenibacillus polymyxa* AF01 against *Neoscytalidium dimidiatum* in pitaya. Front. Microbiol..

[213] Ma X., Gao Y., Li H., Wang D., Li J., Hu X., Huang X., Lin M., Tang Y., Liu Z. (2023). Identification and characterization of biocontrol agent *Lysinibacillus boronitolerans* P42 against *Cerrena unicolor* that causes root rot of arecanut palm. Arch. Microbiol..

[214] Travadon R., Lawrence D.P., Li S., Trouillas F. (2023). Evaluation of biological control agents for the protection of almond pruning wounds against infection by fungal canker pathogens. Phytopathology.

[215] Du B.D., Ngoc D.T.B., Thang N.D., Tuan L.N.A., Thach B.D., Hien N.Q. (2019). Synthesis and in vitro antifungal efficiency of alginate-stabilized Cu_2_O-Cu nanoparticles against *Neoscytalidium dimidiatum* causing brown spot disease on dragon fruit plants (*Hylocereus undatus*). Vietnam J. Chem..

[216] Tuan L.N.A., Du B.D., Ha L.D.T., Dzung L.T.K., Van Phu D., Hien N.Q. (2019). Induction of chitinase and brown spot disease resistance by oligochitosan and nanosilica–oligochitosan in dragon fruit plants. Agric. Res..

[217] Acay H., Yildirim A., Güney I.G., Derviş S. (2022). *Morchella esculenta*-based chitosan bionanocomposites: Evaluation as an antifungal agent. J. Food Process. Preserv..

[218] Hashem A.H., Abdelaziz A.M., Hassanin M.M.H., Al-Askar A.A., AbdElgawad H., Attia M.S. (2023). Potential impacts of clove essential oil nanoemulsion as bio fungicides against *Neoscytalidium* blight disease of *Carum carvi* L.. Agronomy.

[219] Duong N.L., Nguyen V.M., Tran T.A.N., Phan T.D.T., Tran T.B.Y., Do B.L., Phung Anh N., Nguyen T.A.T., Ho T.G., Nguyen T. (2023). Durian shell-mediated simple green synthesis of nanocopper against plant pathogenic fungi. ACS Omega.

[220] Bragard C., Baptista P., Chatzivassiliou E., Di Serio F., Gonthier P., Jaques Miret J.A., Justesen A.F., MacLeod A., Magnusson C.S., Milonas P. (2023). Pest categorisation of *Neoscytalidium dimidiatum*. EFSA J..

