# Supplementary material for: Plant-Associated Neoscytalidium dimidiatum—Taxonomy, Host Range, Epidemiology, Virulence, and Management Strategies: A Comprehensive Review
Source: J Fungi (Basel). 2023 Oct 26;9(11):1048. doi: 10.3390/jof9111048 (PMC10672476; doi:10.3390/jof9111048)
Supplement: Supplementary file 1 [file jof-09-01048-s001.zip › jof-2624917-supplementary.pdf]

Table S1. Data for selected isolates used in phylogenetic analyses, including culture collection number, substrate source, geographic origin, and GenBank accession numbers.

Former taxonomic designation	Culture collection number / Isolate name	Substrate	Country	GenBank accession numbers		
				ITS	*tef1*	*tub2*
	Arp2-D	*Vitis vinifera*	Turkey	MK813852	MK816355	MK816354
	Kale4-C	*Prunus armeniaca*	Turkey	MK788362	MK803351	MK803352
	Nd_Fig01	*Ficus carica*	Turkey	OL304243	OK788657	OK788660
	Nd_Fig02	*Ficus carica*	Turkey	OL304244	OK788658	OK788661
	Nd_Fig03	*Ficus carica*	Turkey	OL304245	OK788659	OK788662
	Ol_Dr04	*Olea europaea*	Turkey	OK416080	OK428813	OK428827
	Ol_Hl02	*Olea europaea*	Turkey	OK416072	OK428805	OK428819
	Ol_Nz19	*Olea europaea*	Turkey	OK416079	OK428812	OK428826
	Nd_Pp01	*Picea pungens*	Turkey	OK643641	OK666381	OK666382
	CBS 125608	Human	Gabon	MH863571	MT592260	MT592752
	CBS 125609	Human	Gabon	MH863572	MT592261	MT592753
	CBS 125610	Human	Gabon	MH863573	MT592262	MT592754
	CBS 125617	Human, toe nail	France	MH863577	MT592264	MT592756
	CBS 125619	Human, foot	France	MT587533	MT592265	MT592757
	CBS 125622	Human, foot	Martinique	MT587537	MT592269	MT592765
	CBS 125623	Human, toe nail	Martinique	MH863579	MT592270	MT592766
	CBS 125695	Human	France	KX464231	KX464764	KX465065
	CBS 125808	Human, foot	Martinique	MH863768	MT592271	MT592767
	CBS 137.77	Cow, bovine foetus	USA	MT587535	MT592267	MT592763
	CBS 145.78	Human, sole of foot	UK	KF531816	KF531795	KF531796
	CBS 251.49	*Juglans regia*	USA	KF531819	KF531797	KF531799
	CBS 312.90	Human, blood	Netherlands	MT587536	MT592268	MT592764
	CBS 499.66	*Mangifera indica*	Mali	KF531820	KF531798	KF531800
	CBS 601.85	Human	USA	MT587538	MT592272	MT592768
	CBS 662.77	Human	–	MT587534	MT592266	MT592758
	CMM 3566	*Jatropha curcas*	Brazil	KF234551	KF226709	KF254935
	CMM 3649	*Jatropha curcas*	Brazil	KF234550	KF226707	KF254934
	COUFAL 0144	*Nopalea cochenillifera*	Brazil	MH251953	MH251961	MH251969
	COUFAL 0145	*Nopalea cochenillifera*	Brazil	MH251954	MH251962	MH251970
	COUFAL 0146	*Nopalea cochenillifera*	Brazil	MH251955	MH251963	MH251971
	DE 1606	*Dioscorea esculenta*	China	KY013660	KY349086	KY349087
Neoscytalidium hylocereum	PSU-HP01	*Hylocereus polyrhizus*	Thailand	LC590859	LC590862	LC647832

Neoscytalidium hylocereum	TSU-HP01	*Hylocereus polyrhizus*	Thailand	LC590860	LC590863	LC647833
Neoscytalidium hylocereum	TSU-HP02	*Hylocereus polyrhizus*	Thailand	LC590861	LC590864	LC647834
Neoscytalidium novaehollandiae	CBS 122070	*Grevillia agrifolia*	Australia	EF585539	EF585579	MT592759
Neoscytalidium novaehollandiae	CBS 122071	*Crotalaria medicaginea*	Australia	EF585540	EF585580	MT592760
Neoscytalidium novaehollandiae	CBS 122072	*Adansonia gibbosa*	Australia	EF585535	EF585581	MT592761
Neoscytalidium novaehollandiae	CBS 122610	*Acacia synchronica*	Australia	EF585536	EF585578	MT592762
Neoscytalidium oculus	IOM 325287	Human, eye	Mexico	MG764431	–	–
Neoscytalidium orchidacearum	MFLUCC 12-0533	Orchid, dead leaves	Thailand	KU179865	–	–
Botryosphaeria dothidea (Outgroup)	CBS 115476	Prunus sp.	Switzerland	AY236949	AY236898	AY236927
